# Invariant surface glycoprotein 65 of *Trypanosoma brucei* is a complement C3 receptor

**DOI:** 10.1038/s41467-022-32728-9

**Published:** 2022-08-29

**Authors:** Olivia J. S. Macleod, Alexander D. Cook, Helena Webb, Mandy Crow, Roisin Burns, Maria Redpath, Stefanie Seisenberger, Camilla E. Trevor, Lori Peacock, Angela Schwede, Nicola Kimblin, Amanda F. Francisco, Julia Pepperl, Steve Rust, Paul Voorheis, Wendy Gibson, Martin C. Taylor, Matthew K. Higgins, Mark Carrington

**Affiliations:** 1 Department of Biochemistry, University of Cambridge, Tennis Court Road, Cambridge, CB2 1QW UK; 2 Department of Biochemistry, University of Oxford, South Parks Road, Oxford, OX1 3QU UK; 3 Kavli Institute for Nanoscience Discovery, Dorothy Crowfoot Hodgkin Building, University of Oxford, South Parks Road, Oxford, OX1 3QU UK; 4 Bristol Veterinary School and School of Biological Sciences, University of Bristol, Bristol, UK; 5 Faculty of Infectious and Tropical Diseases, London School of Hygiene and Tropical Medicine, London, WC1E 7HT UK; 6 Antibody Discovery and Protein Engineering, Biopharmaceuticals R&D, AstraZeneca, Cambridge, UK; 7 School of Biochemistry and Immunology, Trinity Biomedical Sciences Institute, Trinity College Dublin, Dublin, Ireland

**Keywords:** Innate immunity, Parasite immune evasion

## Abstract

African trypanosomes are extracellular pathogens of mammals and are exposed to the adaptive and innate immune systems. Trypanosomes evade the adaptive immune response through antigenic variation, but little is known about how they interact with components of the innate immune response, including complement. Here we demonstrate that an invariant surface glycoprotein, ISG65, is a receptor for complement component 3 (C3). We show how ISG65 binds to the thioester domain of C3b. We also show that C3 contributes to control of trypanosomes during early infection in a mouse model and provide evidence that ISG65 is involved in reducing trypanosome susceptibility to C3-mediated clearance. Deposition of C3b on pathogen surfaces, such as trypanosomes, is a central point in activation of the complement system. In ISG65, trypanosomes have evolved a C3 receptor which diminishes the downstream effects of C3 deposition on the control of infection.

## Abstract (web summary)

Trypanosomes evade the immune response through antigenic variation of a surface coat containing variant surface glycoproteins (VSG). They also express invariant surface glycoproteins (ISGs), which are less well understood. Here, Macleod et al. show that ISG65 of T. brucei is a receptor for complement component 3. They provide the crystal structure of T. brucei ISG65 in complex with complement C3d and show evidence that ISG65 is involved in reducing trypanosome susceptibility to C3-mediated clearance in vivo.

## Introduction

Many eukaryotic pathogens have evolved to establish and maintain proliferative populations within vertebrate hosts, thereby increasing the frequency of opportunities for their transmission by invertebrate vectors. Some pathogens can even maintain a persistent infection in a vertebrate that can last for years. These long-term infections only occur if the pathogen population can survive both the innate and adaptive immune responses.

African trypanosomes are extracellular pathogens that can persist in a human for decades after exposure^1^. They must therefore replicate under direct attack from molecules and cells of the immune response. To achieve this, they have evolved a remarkable cell surface dominated by many copies of a single variant surface glycoprotein (VSG)^2^. This VSG coat both protects the plasma membrane from immunoglobulins^3^ and underpins a system of antigenic variation through which the population avoids clearance by the adaptive immune system^4^. During an infection, antibodies are raised which target the VSGs and the infection persists through a small number of individual cells switching to express antigenically distinct VSGs, which escape and proliferate until they are recognised in turn. Within the VSG coat are found invariant proteins, including nutrient receptors, and a family of invariant surface glycoproteins (ISGs)^5–8^.

In addition to surviving the adaptive immune system, African trypanosomes must also counteract host innate immunity, and in particular the complement system. This system comprises three pathways that centre around the deposition of complement C3b on a cell surface. C3b deposition occurs by a chemical reaction involving a reactive thioester within its thioester domain (TED). This initiates a series of downstream events which can result in cell death^9–11^. A number of different strategies have evolved to regulate or counteract complement. Host cells present complement regulators, such as factor H, which bind C3b and prevent complement activation^12–14^, while a variety of pathogens have evolved receptors that bind complement components and prevent killing^15,16^.

*Trypanosoma brucei* expresses a range of cell surface receptors, including those which mediate interactions with host nutrients, such as transferrin^17–19^ and haptoglobin-haemoglobin^20,21^. It also expresses a cell surface receptor for host factor H^22^, which increases its transmission to tsetse flies. However, despite having been discovered over 30 years ago, and being distributed across the entire cell surface, no ligand had been identified for any of the ISGs. Here we show that ISG65 is a receptor for complement C3 and affects the rate of trypanosome clearance in a mouse infection model.

## Results

### ISG65 binds to complement factor C3b

In *T. brucei*, the ISG65s are encoded by a tandem array of closely related genes on chromosome 2 with the number of genes in one array varying between isolates (Supplementary Fig. 1). These encode type 1 membrane proteins consisting of large ectodomains^23^ and smaller intracellular regions^6^. To understand the function of ISG65, we started by characterising the repertoire of genes from a single isolate. Initial experiments were conducted before the availability of full genome sequences and so the ISG65 locus in *T. brucei* EATRO1125 was characterised from two cosmid clones (Supplementary Fig. 1). Each ISG65 gene is contained within a 3550 bp BamHI fragment and seven such fragments derived from the two cosmids were sequenced, each of which encoded a distinct ISG65 (1125A to G). A sequence comparison of ISG65s from EATRO1125 with those from subsequently available genome sequences^24,25^ (Supplementary Data 1 and Supplementary Fig. 2) indicated that variation is largely confined to blocks within the extracellular domain and revealed that recombination has resulted in a mosaic of N-terminal domains (Supplementary Fig. 2). Subsequent experiments were conducted with ISG65 1125A and G as representatives of the two largest sequence variant groups (Supplementary Fig. 2).

**Fig. 1:**
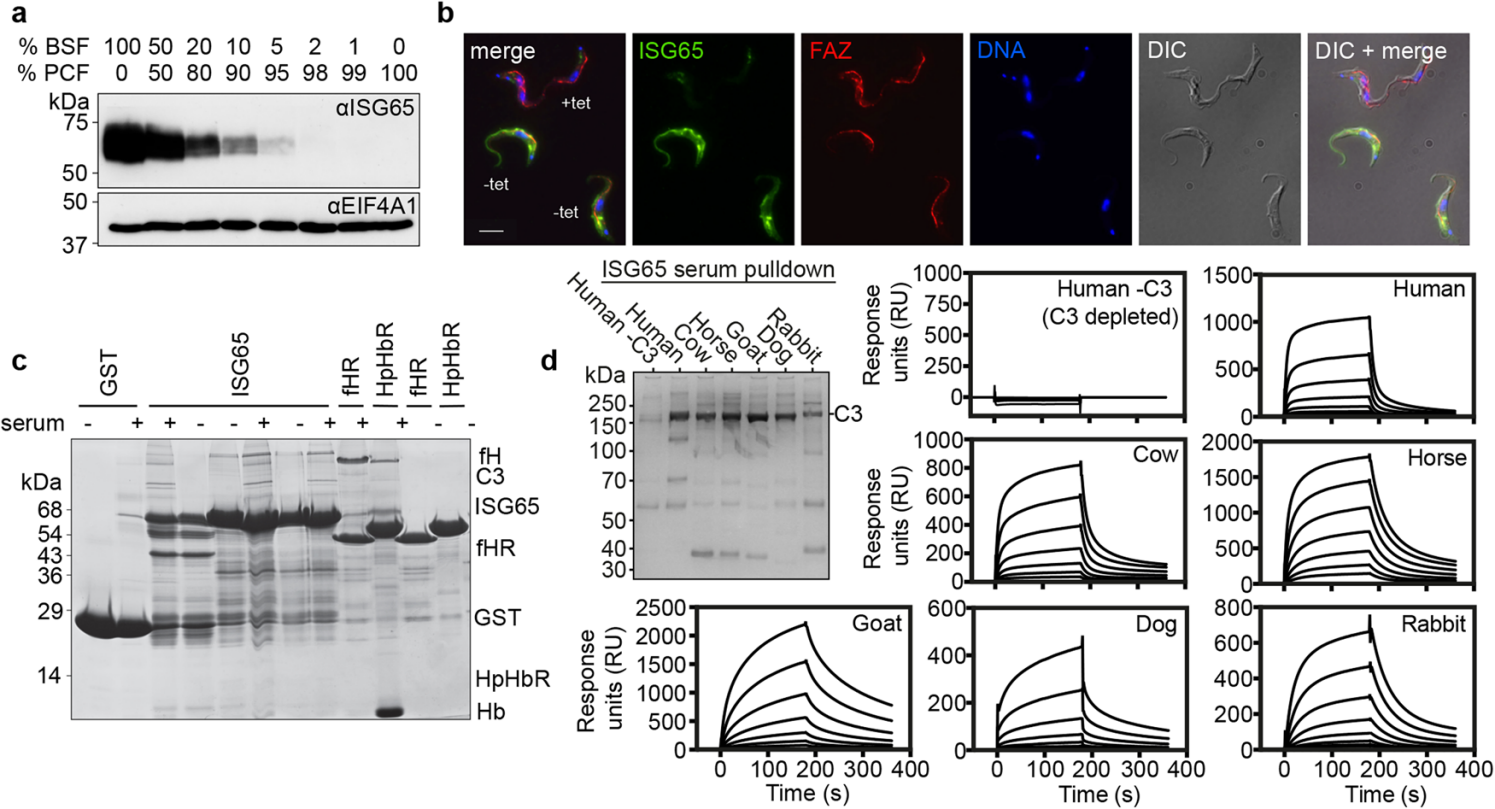
Identification of ISG65 as a receptor for complement factor C3b. **a** A Western blot showing extracts of bloodstream form (BSF) and procyclic form (PCF) trypanosomes, mixed in the proportions indicated above the blot. This blot (*n* = 1) was probed using polyclonal sera against ISG65 (αISG65) (upper panel) and by polyclonal sera against EIF4A1 (αEIF4A1) as a loading control (lower panel). **b** Immunofluorescence images showing ISG65 (green), flagellar marker, FAZ (red), and a DNA marker (blue). Trypanosome cells contain a tet-inducible RNAi knock-down construct for ISG65 and were either incubated with tet (tet+) or not (tet−) before mixing and imaging. The scale bar is 10 μm. Representative images are shown with *n* = 3. **c** An SDS-PAGE gel showing the outcome of pull-down experiments. Beads coated with GST and GST-fusions of ISG65A, the factor H receptor (fHR) and the haptoglobin-haemoglobin receptor (HpHbR) were incubated with bovine serum, before washing and elution. Bands eluted include factor H (fH), complement factor C3 (C3), and haemoglobin (Hb). *n* = 3. **d** Interaction of ISG65G with C3 from different species, showing broad specificity. The gel on the left shows a pull-down where ISG65 was attached to streptavidin beads then incubated with the serum of various mammals, *n* = 1. C3 is the predominant band in all cases except where human serum that was previously depleted of C3 (Human -C3) is used. Surface plasmon resonance traces are also shown for each serum, where ISG65 is immobilised via the C-terminus and binding of serum components from various species is determined.

To confirm earlier findings that ISG65 is expressed on the surfaces of mammalian infective bloodstream form trypanosomes^6,26^, we raised a rabbit antiserum by immunisation with the ectodomain (residues 31–384) of ISG65 1125G, produced from *E. coli*. Western blotting confirmed that ISG65 is detected in bloodstream form cells but not tsetse fly-infective procyclic cells (Fig. 1a). We next visualised ISG65 subcellular localisation using immunofluorescence on cells containing an inducible ISG65 RNAi construct, before and after induction (Fig. 1b). As shown previously^6^, ISG65 was distributed across the cell surface, including across the flagellum, as well as being found in the endosomal compartment.

The presence of ISG65 across the bloodstream trypanosome surface encouraged us to search for a binding partner within bovine serum, employing the same pull-down strategy as used to identify the factor H receptor^22^. We prepared a fusion of residues 22–385 of ISG65 1125A with an N-terminal GST tag expressed in *E. coli*. As positive controls, we also produced both factor H receptor and haptoglobin-haemoglobin receptor with N-terminal GST tags. These receptors were used in pull-down experiments to identify interaction partners in bovine serum. Glutathione beads coupled with GST alone did not purify any components from bovine serum, while the factor H receptor and haptoglobin-haemoglobin receptor purified factor H and haemoglobin, as expected^20,22^ (Fig. 1c). Using the ISG65 fusion to pull-down components from bovine serum led to purification of a protein not observed in the control lanes, with the same protein purified in each of three replicates (Fig. 1c). Analysis by trypsin-digest mass spectrometry revealed this to be bovine complement C3 with 92 identified peptides giving 50% coverage (Supplementary Table 1).

To determine whether ISG65 can bind to C3 from different species, we performed a pull-down assay in which immobilised ISG65 1125G was incubated with serum from six mammals, including humans and livestock (Fig. 1d). While no protein was observed when this was done using human serum depleted of C3, in all other cases ISG65 pulled down a protein of ~180 kDa. These were demonstrated by mass spectrometry to each be C3 from the respective mammal (Supplementary Table 2). Next, we used surface plasmon resonance (SPR) to investigate the kinetics of ISG65 binding to C3. ISG65 1125G with a C-terminal Avi-tag was expressed in cultured mammalian cells and was biotinylated after purification. It was then bound to a streptavidin-coated SPR chip surface and sera from various mammals were flowed over this surface. While no response was observed for human sera depleted of C3, ISG65 bound to a component from each of the six sera with similar kinetics. This indicates that ISG65 shows broad specificity for C3 from a wide range of different mammalian species relevant for trypanosome infection. The appreciable off-rates of these interactions demonstrate that they are reversible and are not due to the formation of a non-specific and irreversible covalent thioester bond between the TED of C3 and ISG65 (Fig. 1d).

### ISG65 binds sequentially to two sites on C3b

We next determined which regions of C3 are involved in the interaction with ISG65. During activation and regulation of the complement cascade, complement C3 undergoes dramatic conformational changes and is processed by multiple cleavage events^12,14,27,28^. The first cleavage removes C3a and the remainder of the molecule undergoes a conformational change to form C3b^9,27–30^. The TED domain of C3b reacts with a cell surface, forming a thioester bond^30^. A subsequent cleavage event then removes the TED domain (now called C3d), leaving it attached to the cell surface, while C3c is released^12,30^.

**Fig. 2:**
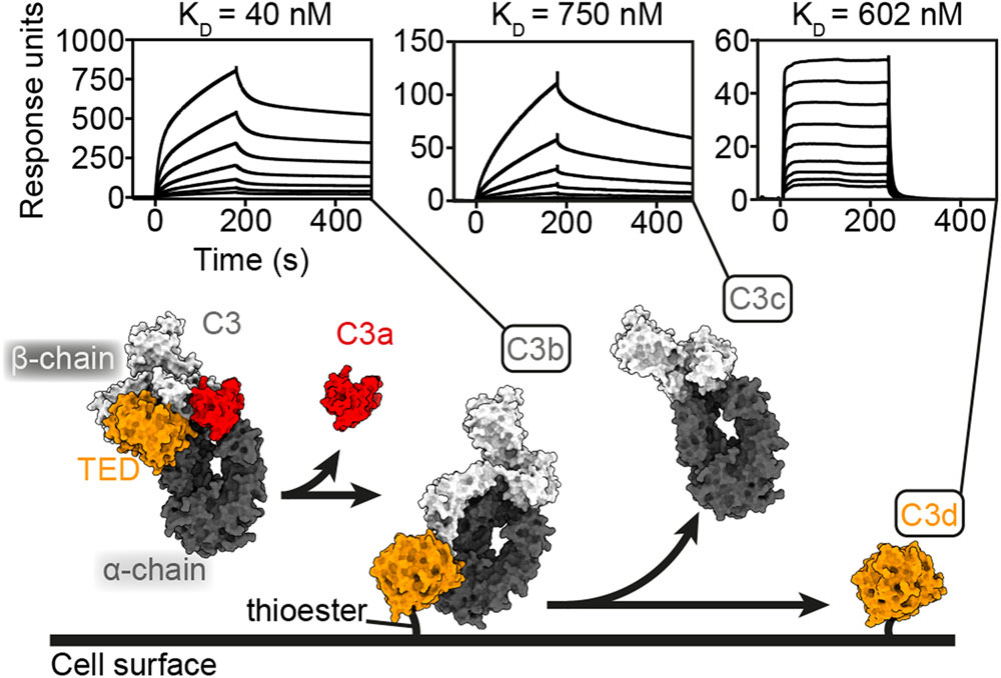
Analysis of the binding of ISG65 to different complement C3b fragments. The lower panel shows a simplified schematic of the deposition of complement C3 on the surface of a pathogen, using surface representations of C3 (PDB ID: 2A73)^30^, C3b (PDB ID: 2I07)^9^, C3c (PDB ID: ﻿2A74)^30^ and C3d (PDB ID: 1C3D)^51^. This is coloured with the α-chain in dark grey, the β-chain in light grey, the TED domain (which becomes C3d) in orange and the domain which is removed to become C3a in red. The upper panels show surface plasmon resonance traces for the binding of C3b, C3c and C3d to immobilised ISG65. These represent two-fold dilution series from an upper concentration of 0.5 μM for C3b and C3c, and 4 μM for C3d, and are representative of three repeats.

To determine at which stage within this process ISG65 might act, we measured the binding of surface-conjugated biotinylated ISG65 to C3b, C3c and C3d, using SPR (Fig. 2). ISG65 bound to each of C3b, C3c and C3d with different affinities and binding kinetics. Binding to C3b was biphasic and did not fit well to a simple single-site binding model (Fig. 2, Supplementary Fig. 3 and Supplementary Table 3). Instead, the data fitted best to a two-state binding model with a dissociation constant of 40 nM. This model describes a complex binding event in which initial binding is followed by a secondary event which increases the overall binding affinity^31^. In contrast, the binding kinetics of ISG65 to either C3c or C3d fitted to a simple single-site binding model, with dissociation constants of 750 nM for C3c and 602 nM for C3d. Despite these similarities in affinity, the binding kinetics were very different, with slow association and dissociation for C3c and faster association and dissociation for C3d. This is consistent with an initial, rapid binding of ISG65 to the TED domain of C3b followed by a secondary event in which ISG65 more slowly interacts with other regions of C3b which are later found in C3c.

Two-state binding to C3b has not been observed for other regulatory C3 receptors. ﻿*S. aureus* Efb-C^32^, Sbi^33^, and Ehp^34^ all bind to the TED domain in a 1:1 binding mode and the complement regulator Factor H binds to C3b in a 1:1 fashion^35,36^. The functional consequences of ISG65 binding both to the TED domain and the C3c region of C3b are yet to be discovered but could result from a need to independently interact with C3b and also the C3d fragment that remains bound to the trypanosome surface after C3b cleavage. Alternatively, binding to the TED and the C3c region of C3b simultaneously may be essential for ISG65 to interfere with C3 function.

### The structural basis for C3d binding by ISG65

**Fig. 3:**
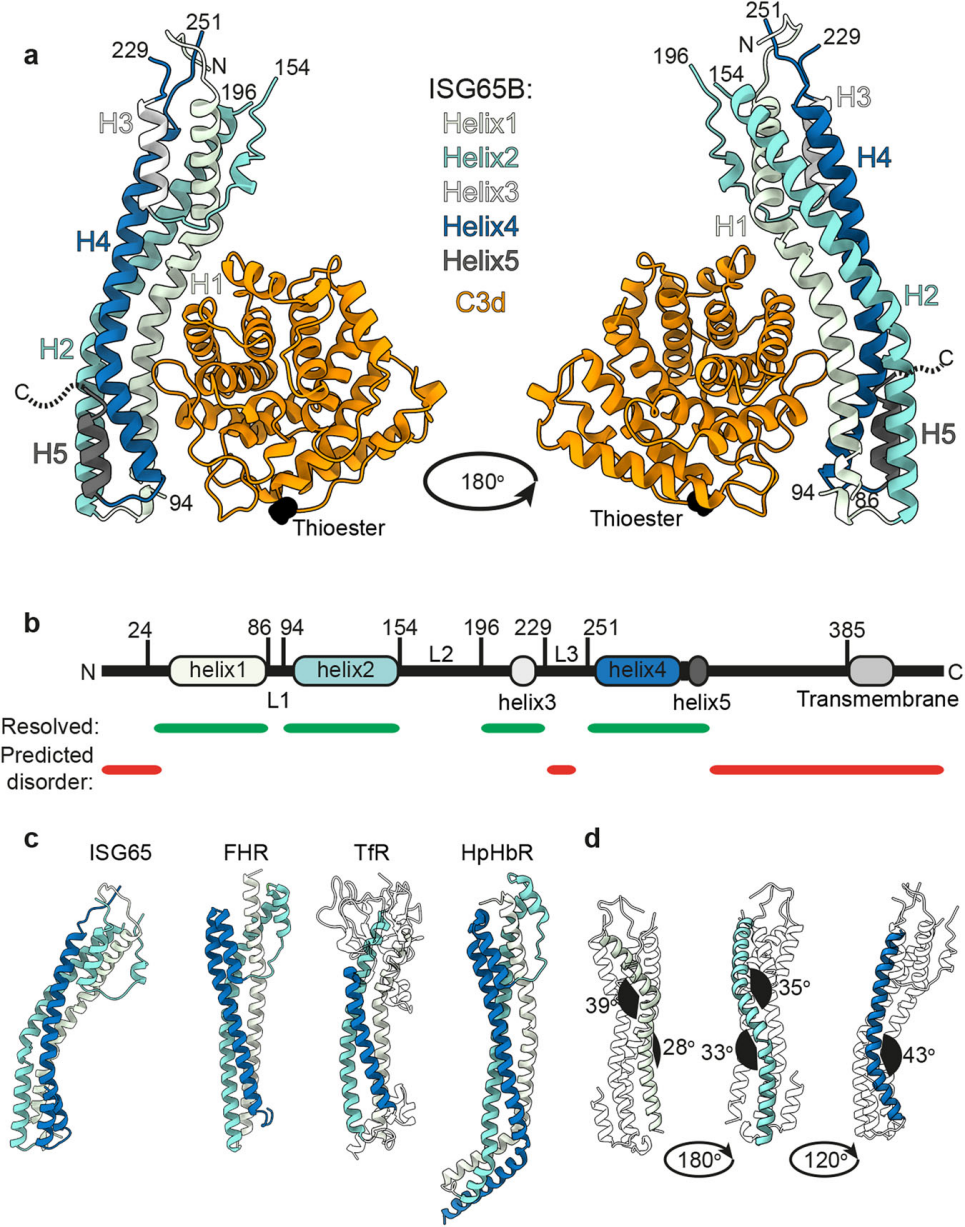
Structure of *T. brucei* ISG65G receptor bound to complex C3d. **a** The structure of ISG65 in complex with C3d. The helices of ISG65 are each coloured differently, with core helices 1, 2, and 4 shown in light green, turquoise, and blue, while shorter ISG65 helices 3 and 5 are shown in light grey and dark grey. All helices are labelled H1–H5. C3d is shown in orange, with the location of the thioester bond-forming cysteine highlighted black. N and C indicate the termini of ISG65. **b** A schematic showing the features of ISG65. The location of helices 1–5 are shown, using the same colours as **a**, while the transmembrane helix, predicted using TMHMM, is light grey. Green bars show regions of ISG65 that were resolved in the crystal structure, and red bars show regions predicted to be disordered by AUCpreD^65^. The three loops in between helices, which are largely unresolved, are labelled L1–L3. **c** Comparison of the structure of ISG65 with equivalent views of the *T. brucei* factor H receptor, FHR^22^, the transferrin receptor, TfR^17^ and the haptoglobin-haemoglobin receptor, HpHbR^20^. **d** Different views of the ISG65 structure with helix 1 (light green), helix 2 (turquoise), and helix 3 (blue) highlighted to show the kinks that give rise to the curved structure of ISG65. The angles of each kink were measured using Chimera^66,67^, and are indicated in black.

We next determined the structure of ISG65 in complex with C3d. We expressed C3d in *E. coli* and combined it with the ectodomain of ISG65 1125G expressed in CHO cells. Crystals formed and the structure was solved to 2.6 Å using molecular replacement (Fig. 3a and Supplementary Table 4). Two copies of the complex were present in the asymmetric unit, with each receptor contacting three different C3d molecules within the crystal lattice, with surface areas of 814, 553, and 530 Å^2^ for interfaces 1, 2, and 3 respectively. To determine which of these putative complexes is physiologically relevant, and which is due to crystal packing, we made C3d mutants at each interface, aiming to disrupt complex formation. We generated E1110I and P1114R mutants to disrupt interface 1, I1125N to disrupt interface 2 and L1046R to disrupt interface 3. C3d and mutants all had similar CD spectra, indicating no effect of these mutations on overall C3d structure (Supplementary Fig. 4). C3d mutations at interfaces 2 and 3 had little effect on affinity for ISG65 when compared with wild-type C3d, whilst both mutations at interface 1 had a large effect on binding to ISG65, with P1114R ablating binding (Supplementary Fig. 4). This allowed us to define interface 1 as the physiological interaction interface for ISG65 binding.

While ISG65 residues 24–385 were included in the construct used for crystallisation, just ~60% of the chain was resolved in the electron density (residues 27–86, 94–154, 196–229, and 251–315). The ordered core of the ISG65 N-terminal domain consists primarily of a three-helical bundle, with helices 1, 2, and 4 lying approximately parallel to each other, creating an elongated architecture (Fig. 3). In between helices 2 and 4 is a structured loop which contains the shorter helix 3. Two loops which are almost entirely unresolved (L2 and L3, residues 154–196 and 226–256 respectively) also emerge from this end of the molecule (Fig. 3). At the other end, short helix 5 forms a hairpin with helix 4, placing the C-terminus along the helical bundle, opposite where C3d binds. A total of 73 residues link this residue with the transmembrane helix, and whilst these residues were present in the construct subjected to crystallisation trials, they were not resolved and are presumed to be disordered. The position of helix 5 would allow this disordered region to project at an angle from the long-axis of ISG65, most likely leading to a flexible attachment of the C3d binding domain to the membrane anchor (Supplementary Fig. 5). While ISG65 shares many features with other *T. brucei* three-helical bundle surface receptors^17,20,22,37^ it shows a much greater degree of curvature (Fig. 3c), due to two kinks in helices 1 and 2, and one larger kink in helix 3 (Fig. 3d).

The curvature of ISG65 produces a concave surface into which C3d binds (Fig. 3a). This interface is mediated by thirteen hydrogen bonds, three salt bridges and 24 residue-residue contacts (Supplementary Table 5), involving a region of low B-factor (Supplementary Fig. 5a). To further validate this model, we collected SAXS data for the ISG65-C3d complex, and reconstructed a 3D volume (Supplementary Fig. 5b, c). The ISG65-C3d crystal structure fitted well into the SAXS density, and also revealed density unaccounted for by the crystal structure. The extra density extends from the end containing the N-terminus of ISG65 and is most likely attributable to L2. The location of the ISG65 binding site does not overlap with the thioester of C3d, which allows ISG65 to bind to C3d that is covalently coupled on the trypanosome surface. In addition, the observed mode of C3d binding is consistent with ISG65 being able to bind to the TED domain of C3 or C3b (Supplementary Fig. 5d).

### Complement C3 is necessary for control of the initial wave of a trypanosome infection and ISG65 delays trypanosome clearance

To test the role of ISG65 in infection, we deleted the entire locus containing the ISG65 genes. The starting cell line was *T. brucei* EATRO1125 pleomorphic bloodstream form cells^38^ which express far red-shifted luciferase, Cas9 and T7 RNA polymerase transgenes. The ISG65 locus was deleted using guide RNAs which directed cleavage at unique sequences either side of the locus and deletions were repaired with blasticidin and G418 resistance markers. Deletion of the ISG65 genes was confirmed by genome sequencing and had no effect on the proliferation rate of the cell line in culture (Supplementary Fig. 6).

**Fig. 4:**
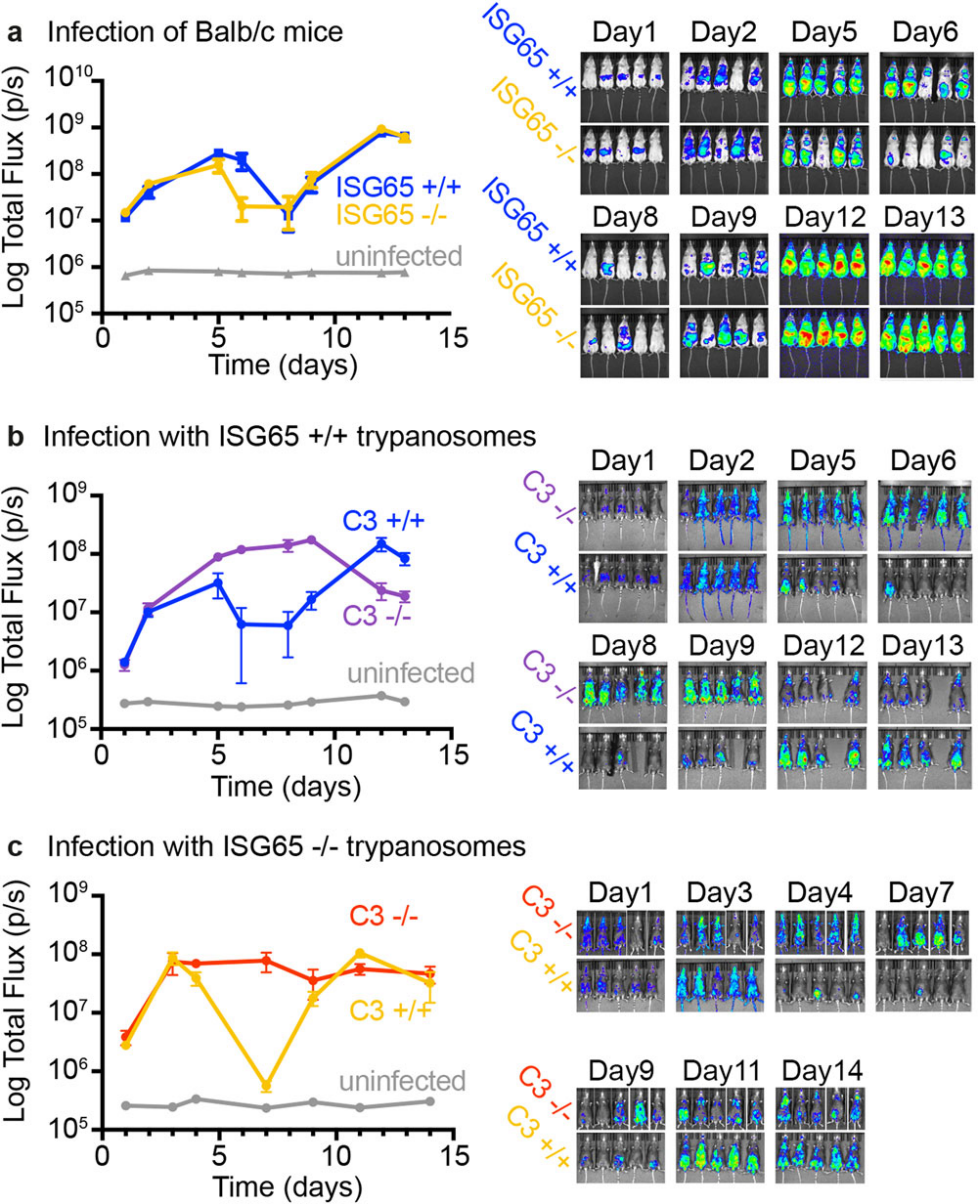
Assessment of the effect of ISG65 and C3 knock-out on trypanosome growth in mice. **a** The effect of ISG65 knock-out on trypanosome infections in Balb/c mice. Five mice were infected with bioluminescent ISG65+/+ (blue, *n* = 5 animals) or ISG65−/− (orange, *n* = 5 animals) *T. brucei* cell lines. Two uninfected mice were used as controls for basal bioluminescence (grey, *n* = 2 animals). Trypanosome burden was measured by imaging bioluminescence over time. **b** The effect of C3 knock-out on trypanosome infections in C57BL/6 mice. Bioluminescent ISG65+/+ *T. brucei* were used to infect five C57BL/6 mice (blue, *n* = 5 animals) and five C57BL/6 mice lacking C3 (purple, *n* = 5 animals). Two uninfected mice were used as controls for basal bioluminescence (grey, *n* = 2 animals). Trypanosome burden was measured by imaging bioluminescence over time. **c** The effect of C3 knock-out on ISG65−/− trypanosome infections in C57BL/6 mice. Bioluminescent ISG65−/− *T. brucei* were used to infect five C57BL/6 mice (orange, *n* = 5 animals) and five C57BL/6 mice lacking C3 (red, *n* = 5 animals). Two uninfected mice were used as controls for basal bioluminescence (grey, *n* = 2 animals). Trypanosome burden was measured by imaging bioluminescence over time. Individual mice are seen in Supplementary Fig. 7. The data presented in **a** and **b** were conducted concurrently, while that presented in **c** was conducted subsequently and should not be directly compared with the data in **a** and **b**. Points represent the mean and error bars the standard deviation.

We next assessed the effect of ISG65 deletion on growth in a mammalian host using the standard BALB/c mouse model. Groups of five mice were infected with ISG65+/+ or ISG65−/− trypanosomes. Parasite burden was monitored over 13 days by measuring bioluminescence produced by the luciferase transgene (Fig. 4a and Supplementary Fig. 7a). In each infected mouse, we observed two waves of infection. A first wave peaked around 5 days post infection and then was partly controlled, resulting in reduced parasite burden. Around 8 days post infection, a second wave of infection started, most likely initiated by trypanosomes which had undergone antigenic variation^39^. The primary difference was in the rate of control of the first wave of infection, with a more rapid decrease in parasite burden seen from day 5 in the ISG65−/− trypanosomes than in ISG65+/+ trypanosomes.

To investigate whether complement C3 is necessary for control of parasite burden in the first wave of infection, we infected mice lacking C3 with ISG65+/+ or ISG65−/− trypanosomes. Mice lacking C3 were in a C57BL/6 background and so we also infected wild-type C57BL/6 as a control. ISG65+/+ trypanosomes were used to infect groups of either five C57BL/6 mice or five mice lacking C3. Parasite burden was monitored by measuring bioluminescence (Fig. 4b and Supplementary Fig. 7b). In mice with C3, we again observed two waves of infection, with reduction of parasite burden starting around day 5. In contrast, mice lacking C3 did not control the infection and parasite burden remained high (Fig. 4b). When an equivalent experiment was subsequently conducted using ISG65−/− trypanosomes, we observed a similar effect, with mice lacking C3 not able to control the infection (Fig. 4c and Supplementary Fig. 7c).

These data combine to suggest that complement-mediated processes, requiring complement C3, shorten the first wave of infection. They also show that ISG65, the C3b receptor, reduces the susceptibility of trypanosomes to C3-mediated clearance and that parasite burden decreases more rapidly in its absence.

## Discussion

Three distinct, but overlapping pathways can trigger the effects of complement in infection. In the classical pathway, antibodies mediate recruitment of complement C3b. In the lectin pathway, this is mediated by recognition of pathogen surface oligosaccharides. In contrast, the alternative pathway results from stochastic activation of C3, resulting in deposition of C3b. Therefore, the alternative and lectin pathways can operate from the start of infection, while the classical pathway requires an antibody response. In the mouse infection models used here, the initial increase in parasite burden occurs at an equivalent rate in both C3+/+ and C3−/− mice, which suggests that the alternative and lectin-based pathways are not controlling infection. The decrease in parasite burden in the latter part of the first wave of infection is therefore mostly likely due to the induction of trypanosome-targeting immunoglobulins. The requirement for complement C3 for this decrease indicates that it results from the classical pathway of complement. Our finding that ISG65 slows this control of parasite burden (Fig. 4a) therefore suggests that it controls the function of C3b deposited on trypanosome surfaces through the classical pathway.

While we see no evidence for the role of the alternative pathway of complement in controlling the parasite burden of a virulent trypanosome strain during mouse infection, we do not rule it out in infections in which trypanosomes are less virulent, or less adapted to growth in mice. Indeed, studies conducted in vitro have shown that the alternative pathway can be initiated on trypanosome surfaces^40,41^. The incubation of trypanosomes with serum results in conjugation of C3b to the VSG coat^41^. To assess how far the complement cascade proceeds, deposition of complement factors, which occurs after C3b conjugation, was investigated. C3b deposition on trypanosomes was enhanced by factors B and D, and surface bound factor B was observed, suggesting alternative pathway activation^40^. However, C5 and C9 were not detected on the surfaces of these trypanosomes, suggesting that the membrane attack complex does not form and that the complement cascade does not proceed beyond C3 activation^40^. This raises the question of what features of the trypanosome surface limit progress of the complement cascade.

In this study, we show that ISG65, which is distributed over the entire trypanosome surface, is a complement C3b receptor. As C3b is a core component of each of the alternative, classical and lectin pathways of complement, ISG65 has the potential to reduce complement-mediated killing through each of these pathways. Indeed, we observed that the absence of ISG65 leads to more rapid control of the first wave of infection in a mammalian infection model, suggesting that the receptor is part of a redundant system that has evolved to reduce the effects of complement. What role may it play in blocking the pathways of complement?

It is likely that multiple mechanisms will converge to allow ISG65 to contribute to resistance to complement-mediated killing. One mechanism, which also reduces detection of trypanosomes by immunoglobulins, is rapid endocytosis and recycling of the cell surface. Antibodies attached to VSGs are swept towards the posterior of trypanosomes by hydrodynamic forces conferred by a swimming motion^42^. These antibodies then enter the flagella pocket, where they are endocytosed and degraded in the lysosome. It seems likely that a similar mechanism will contribute to clearance of VSG-conjugated C3b, reducing accumulation of C3b on the cell surface. Nevertheless, at any point in time, there will still be surface deposited C3b. A receptor which prevents this surface pool from triggering the later steps of the complement cascade will therefore contribute to trypanosome survival. How would such a receptor work?

For the receptor to block downstream events of the complement cascade, it must be able to bind C3b that is conjugated to any of the exposed surfaces of the VSG. This presents an access challenge to the receptor, as much of the C3b is likely to be deposited on the most exposed, membrane distal part of the VSG. However, the linker which joins the C3d-binding domain of ISG65 to the transmembrane helix is 73 residues long and is predicted to be flexible. Such a linker would, if fully extended, reach ~260 Å from the cell surface, sufficient to extend above the ~155 Å thick VSG coat. This would enable receptors to bind to C3b attached to any part of the trypanosome surface. With ISG65 distributed across the entire surface, at a density of ~70,000 copies per cell, these receptors can then shield deposited C3b from other complement components it as it is swept towards the flagellar pocket and its subsequent destruction.

Comparison of ISG65 with the factor H receptor^22^ shows them to operate in different developmental stages of the trypanosome life cycle. The factor H receptor is only expressed in bloodstream form cells which have arrested as an adaption for transmission to tsetse flies^22^ whereas ISG65 is expressed in both proliferating and arrested bloodstream forms. While ISG65 is no longer expressed in proliferating procyclic form trypanosomes in the tsetse fly midgut, factor H receptor expression increases here, to counter complement in blood meals^22^. As a result, ISG65 is part of a multicomponent system that reduces the effectiveness of complement during growth in mammalian blood, while the factor H receptor affects transmission to tsetse flies^22^. This highlights two very distinct mechanisms by which African trypanosomes reduce destruction by complement during different phases of their life cycle. There remain at least six further families of ISGs with unknown function, and we anticipate that some of these will also contribute to survival under the onslaught of complement.

## Methods

### Expression and purification of trypanosome receptors

To produce ISG65 in *E. coli*, in order to raise antisera, ISG65 1125G (residues 31–384) was expressed from a pET21b vector with a C-terminal His_6_ tag as previously described^43^. Protein was expressed in *E. coli* BL21 (DE3) pRIPL, through induction at 37 °C for 3 h with 0.02% (w/v) isopropyl β-D-1-thiogalactopyranoside (IPTG). Following cell lysis, soluble protein was purified with nickel affinity chromatography and buffer exchanged into phosphate-buffered saline (PBS).

N-terminally tagged GST fusion proteins, used in pull-down experiments in Fig. 1c, were expressed from a pGEX-KG vector, modified to contain a GSSGGGG linker between tag and receptor. The construct used to express GST-factor H receptor (FHR) has been described before^22^. The gene encoding residues 23–274 of the *T. congolense* haptoglobin-haemoglobin receptor (TcHpHbR) was PCR amplified from *T. congolense* IL3000 genomic DNA. ISG65 1125A (residues 22–385) was codon optimised for *E. coli* expression (Invitrogen). Both were inserted into the pGEX-KG vector, with both containing a Tobacco Etch Virus (TEV) cleavage site between the GST tag and the receptor sequence. GST fusion proteins were expressed in *E. coli* BL21, through induction at 37 °C for 3 h with 0.02% (w/v) IPTG. Following cell lysis, soluble protein was purified with glutathione-sepharose affinity chromatography and was buffer exchanged into PBS.

To produce ISG65 in Chinese Hamster Ovary (CHO) cells or HEK293 cells, for structural and biophysical studies, ISG65 1125G (residues 24–385) was codon optimised for expression in mammalian cells and was cloned into a pDest12 vector. Protein expression was driven by a cytomegalovirus promoter and a CD33 signal peptide was used for secretion. The receptor sequence was followed by a flexible linker (GSGSGSASG), an AviTag, and a His_10_ tag. G22 CHO cells were grown in serum-free CCM8 medium (SAFC) and 500 ml of culture was transfected with expression plasmid as described^22^. Cells were fed on days 1, 3 and 6 with 3.3% F9 and 0.2% F10 (AstraZeneca). Secreted protein was captured from culture supernatant by nickel affinity chromatography, followed by buffer exchange into PBS. HEK293F cells were grown in FreeStyle F17 medium (Thermo Fisher) and transfected using polyethyleneimine at a cell density of 2.5 × 10^6^ cells. The culture supernatant was harvested 6 days after transfection, and secreted ISG65 was purified using Ni Sepharose excel resin (Cytiva), followed by size exclusion chromatography with Superdex 75 300/10 GL.

### Protein modification

Proteins containing an AviTag were biotinylated in vitro by mixing purified protein (30 μM) with BirA ligase (0.5 μM), ATP (5000 μM) and biotin (300 μM). The mixture was incubated overnight at room temperature, followed by buffer exchange to remove free biotin.

Glycan removal was performed using PNGase F (NEB) under native conditions and incubation at 37 °C for 4 h. For crystallography and C3d SPR, ISG65G was expressed with kifunensine, and glycans were removed by incubation with Endo H_f_ (NEB) for 1 h at 37 °C.

### Protein electrophoresis and visualisation

Protein samples were prepared and analysed using by SDS-PAGE and standard methods. Western blotting used Immobilon-P membrane (Millipore) and standard methods. Rabbit ISG65 antiserum was generated against recombinant ISG65 1125G produced in *E. coli* (Covalab) and used as primary antibody. Anti-eIF4A^44^ was used as loading control. Donkey or goat anti-rabbit horseradish peroxidase conjugates were used as secondary antibody.

### Source of C3 derived fragments

Human complement C3b and C3c were purchased (Complement Technology). Complement C3d was expressed in *E. coli*. DNA encoding residues 996–1303 of *H. sapiens* C3 containing a C1010A mutation was codon optimised for *E. coli* expression (Invitrogen), and was cloned into the pET15b expression vector (Novagen) using Gibson Assembly (NEB). C3d was expressed in *E. coli* by induction with 0.25 mM IPTG for 18 h at 18 °C. Following cell lysis, C3d was purified from soluble material by Nickel affinity chromatography, then by size exclusion chromatography on a Superdex 200 in 20 mM HEPES pH 7.4, 150 mM NaCl. E1110I, P1114R, I1125N, and L1046R mutants were purified in the same way.

### Generation of RNAi cell line

The approach for hairpin RNAi was as described^45^. The hairpin RNAi plasmid p2950 contained an inverted repeat of the 3’ 335 nucleotides of the ISG65 open reading frame separated by a 725 bp stuffer fragment.

### Immunofluorescence

The ISG65 RNAi cell line was grown without or with tetracycline for 24 h. In total, 1 × 10^7^ cells from each were harvested and resuspended in 1 ml HMI-9 and fixed by the addition of an equal volume of 8% paraformaldehyde in PBS. After 1 h, 10 ml PBS was added and the cells collected by centrifugation and resuspended in 1 ml PBS. In total, 50 μl aliquots were placed on poly-lysine slides and cells were allowed to settle for 1 h. Post-fixation permeabilisation involved a 5 min incubation in 0.1% Triton X-100 in PBS. The primary antibody was a mixture of affinity purified anti-ISG65 and a mouse monoclonal anti-FAZ (a kind gift of Keith Gull, University of Oxford). Secondary antibodies were donkey anti-rabbit IgG AlexaFluor 488 and goat anti-mouse IgG AlexaFluor 568. Hoechst 33258 was used to visualise DNA. Microscopy was performed using a Zeiss Axioimager M1; images were recorded using the Axiovision software (Zeiss) and the same settings were used for all samples within a set of experiments. Images were imported into Adobe Photoshop for figure preparation.

### Pull-down experiments

Pull-downs used to identify C3 (Fig. 1c) were performed as previously described^22^. In brief, glutathione-sepharose 4B (GE Healthcare, 140 μl slurry/pull-down) was incubated with GST-tagged protein (1 mg/pull-down) for 30 min, washed with PBS and incubated with bovine serum (Sigma-Aldrich, 1 ml/pull-down) or a PBS control for 60 min. ISG65 E1125A produced in *E. coli* was used in these experiments. The beads were rapidly washed with three PBS washes with a total time of 5 min. Bound protein was eluted by adding SDS-polyacrylamide gel electrophoresis sample buffer and heating at 50 °C. The pull-downs using GST-ISG65A as bait were performed in three replicates. Pull-downs used to identify C3 from different mammalian species (Fig. 1d) were performed by binding avi-tagged ISG65 E1125G to Pierce Streptavidin Agarose beads (Thermo Fisher). Serum from various mammalian species (Sigma) was added to ISG65-streptavidin beads via gravity flow. The beads were then washed three times with HBS pH 7.4, and C3 bound to ISG65 eluted with 0.1 M sodium acetate pH 4, 500 mM NaCl.

### Mass spectrometry

Pull-down samples from Fig. 1c were run by SDS-PAGE and unique bands for ISG65 E1125A were excised from one replicate, as was the corresponding region from the GST-FHR control pull-down. Samples were trypsin-digested and analysed by liquid chromatography-tandem MS (LC-MS/MS) system using electrospray ionisation-quadropole-time of flight (ESI-QUADTOF) by the Cambridge Centre for Proteomics, using standard protocols. Searches were performed against a database that contained sequences from the UniProt *Bos taurus* database (taxID 9913), the cRAP database (with *B. taurus* removed to avoid duplicates), as well as sequences for trypsin, LysC, and benzonase (total = 32,282 sequences) and were analysed by Mascot. Label-free quantitation was performed using the online label-free spectral normalised index quantitation software (SINQ) tool^46^.

Pull-down samples from Fig. 1d were analysed by SDS-PAGE and the most intense band for each species was excised. The samples were trypsin-digested and recovered peptides were analysed by LC-MS/MS. Peptides were separated by nano liquid chromatography (Thermo Scientific Easy-nLC 1000) coupled in-line to a Q Exactive mass spectrometer equipped with an Easy-Spray source (Thermo Fischer Scientific). Peptides were trapped onto a C18 PepMac100 precolumn (Thermo Fischer Scientific) using 0.1% Formic acid. The peptides were further separated onto an Easy-Spray RSLC C18 column (Thermo Fischer Scientific) using a linear gradient from 15 to 35% of 0.1% formic acid in acetonitrile, at a flow rate 200 nl/min. Full-scan MS spectra were acquired in the Orbitrap. The ten most intense peaks were selected for higher-energy collision dissociation fragmentation. For protein identification, tandem mass spectra were searched using SEQUEST HT within Proteome discoverer PD1.4 (Thermo Fischer Scientific, version 1.4.0.288) against sequence databases containing protein entries from organisms of interest and common contaminants *(*Human*: Homo Sapiens*, Uniprot release 2022-01-24, 20549 protein entries*;* Bovine*: Bos Taurus*, Uniprot release 2022-01-28, 6233 protein entries; Horse: *Equus Caballus*, Uniprot release 2022-01-27, 44765 protein entries; Goat: *Capra Hircus*, Uniprot release 2022-01-27, 35765 protein entries; Rabbit: *Oryctolagus cuniculus*, Uniprot release 2022-01-27, 1281 protein entries*)*.

### Surface plasmon resonance

All SPR experiments were performed on a BIAcore T200 (Cytiva). Binding of human complement C3b and mammalian sera (Sigma-Aldrich) was performed by immobilising biotinylated ISG65G produced in CHO cells (~800 RU) to a streptavidin-coated chip (Series S Sensor Chip SA, Cytiva). C3b and C3c were run from 500 nM in two-fold dilutions to 15.6 nM and sera were diluted 1:10, 1:20, 1:40 and 1:80. Measurements were performed at 30 μl/min at 25 °C in 20 mM HEPES pH 7.4, 150 mM NaCl, 0.005% TWEEN-20, with 180 s injection and 300 s dissociation. Regeneration was performed using 0.1 M glycine-HCl pH 2.5. To study the binding of serum components from different mammalian species to ISG65, ISG65G produced in HEK293 cells was bound to a streptavidin-coated chip (~1300 RU), (Series S chip CM5, Cytiva, with streptavidin coupled to ~1450 RU). Sera from various mammals was flowed over the chip in two-fold dilutions from 8 mg/ml, at a flow rate of 30 μl/min at 25 °C in 20 mM HEPES pH 7.4, 150 mM NaCl, 0.005 % TWEEN-20. Regeneration was performed using 0.1 M sodium acetate pH 4, 500 mM NaCl.

To measure binding of C3d and mutants, ISG65 1125G was immobilised by amine coupling (345 RU) to a CM5 capture chip (Cytiva). To assess the affinity of C3d for ISG65G, C3d was run from 4 μM in a two-fold serial dilutions. To assess how C3d mutants impacted binding, C3d and mutants were flowed at a fixed concentration. C3d and mutants were flowed at 30 μl/min, with 240 s injection, 300 s dissociation at 25 °C, in 20 mM HEPES pH 7.4, 300 mM NaCl, 0.05 % TWEEN-20. Data collected for binding to a blank flow path was used to subtract non-specific binding. Buffer was also flowed over immobilised ISG65 under the same conditions and the response was subtracted to correct for signal drift. All SPR experiments were performed in triplicate. Binding responses were obtained using BIAevaluation software v1.0, enabling determination of affinity (K_D_). Data for C3c and C3d were fit to the 1:1 Langmuir model, yielding k_on_ and k_off_ kinetic parameters. Data for C3b was fit to a two-state reaction model, yielding k_on1_, k_on2_, k_off1_, and k_off2_ kinetic parameters.

### Crystallisation, data collection and structure determination

ISG65G and C3d were mixed in a 1:1 ratio and the complex was purified by size exclusion chromatography using a superdex 200 (Cytiva) in 10 mM HEPES pH 7.4, 50 mM NaCl and was concentrated to 13.4 mg/ml. Complex was mixed at a 1:1:0.5 ratio with crystallisation buffer (0.1 M MES pH 6, 0.2 M MgCl_2_, 20% PEG 6000, Molecular Dimensions) and silver bullet HR2-996-55 (Hampton Research) in a 250 nl drop. Crystals grew by vapour diffusion in sitting drops after 5 days at 18 °C.

Diffraction data were collected on the MASSIF1 beamline at ESRF using a Pilatus3_2M detector (Dectris) at a wavelength of 0.9655 Å. Data was indexed and integrated using Dials v3.3^47^ then scaled with AIMLESS v0.7.7 in CCP4i2 v1.0.2^48,49^, yielding a dataset extending to 2.6 Å. The structure was solved by molecular replacement using PHASER v2.8.3^50^, with two copies of C3d (PDB ID: 1C3D^51^) used as a search model. ISG65 was then built de novo using Coot 0.9.4^52^, and the structure was refined iteratively with BUSTER v2.1^53^ and Coot. ChimeraX was used for visualisation.

### Small-angle X-ray scattering analysis

Size exclusion chromatography-coupled SAXS data of ISG65G-C3d complex was collected at the B21 beamline at Diamond Light Source^54^ using a wavelength of 1 Å. 12.6 mg/ml ISG65G-C3d was run on a Shodex KW-403 in 10 mM HEPES pH 7.4, 50 mM NaCl at a flow rate of 0.16 ml/min, and SAXS data collected at 1 frame/s. ATSAS v3.0.3^55^ was used to automatically select frames containing scattering of ISG65G-C3d, and also frames containing only scattering caused by buffer, which was subtracted from ISG65G-C3d frames. GNOM in ATSAS was used to calculate a fit to the scattering data and was calculate the pair-distribution function using an *R*_max_ of 145 Å. An ab initio electron density map was then calculated using DENSS v1.6^56^, with a final map calculated by averaging 20 independent runs.

### Circular dichroism

CD spectra were recorded between 190 to 260 nm on a Jasco J-815 spectropolarimeter with a path length of 1 mm. C3d-WT, E1110I, P1114R, I1125N, and L1046R were measured at 0.24, 0.25, 0.25, 0.24, 0.21 mg/ml respectively in 10 mM HEPES, 150 mM NaF pH 7.4. Ten runs were recorded and averaged for each spectra, then converted to mean residue ellipticity using CAPITO^57^.

### Knock-out cell lines

*T. brucei* EATRO1125 bloodstream forms^58^ were maintained in standard culture conditions between 1 × 10^5^ and 1 × 10^6^ cells/ml and experiments were performed when the cells were in mid-log phase. Transfections were performed using standard procedures and transfected clones were selected using 10 μg/ml blasticidin, 1 μg/ml puromycin, 25 μg/ml hygromycin or 10 μg/ml G418 as appropriate.

Deletion of the ISG65 gene array in the EATRO1125 cell line was performed using clustered regularly interspaced short palindromic repeats (CRISPR)-associated gene 9 (Cas9) gene editing. The parental blood stream form cell line was first modified by transfection with a construct containing a red-shifted luciferase^59^. Next, a luciferase-expressing clone was further modified by transfection with a construct containing Cas9, T7 RNA polymerase transgenes enabling genome editing^60^. Primers were designed using the LeishGEdit online database (leishGEdit.net). The resultant cell line was then transfected with PCR products to perform ISG65 knock-out as described; two rounds were need to delete first one and then the second allele.

### Genome sequencing

Genomic DNA was prepared from cell lines using Qiagen DNeasy Blood and Tissue kit. DNA was sequenced using a 150 bp library paired-end approach at the Beijing Genomics Institute. Quality filtering was performed using trimmomatic^61^ using the settings: LEADING:10 TRAILING:10 SLIDINGWINDOW:5:15 MINLEN:50. The quality filtered paired-end reads were then mapped to the *T. brucei* TREU927 genome^24^ (v46 from www.tritrypdb.org) using Bowtie2^62^ with default settings on the Galaxy platform^63^ and visualised using Artemis^64^.

### Analysis of trypanosome infection in a mouse model

All experiments were performed using female BALB/c (6–8 weeks of age), C57BL/6J or C57BL/6J C3−/− (Jackson mouse stock no. 003641) (both 8–9 weeks of age) mice purchased from Charles River (UK). They were maintained in individually ventilated cages, under specific pathogen-free conditions, with a 12-h light/dark cycle, ambient temperature of 19–24 °C, 45–65% humidity, and were provided with food and water ad libitum. Research was carried out under UK Home Office project licenses PPL 70/8207 and P9AEE04E4 (protocol 04), with approval of the LSHTM Animal Welfare and Ethical Review Board, and in accordance with the UK Animals (Scientific Procedures) Act 1986 (ASPA). Mice were infected intraperitoneally with 1 × 10^6^ BSF trypomastigotes of *T. brucei* EATRO1125 Luc ISG65+/+ or EATRO1125 Luc ISG65−/−. Five mice were infected for each group and two mice remained uninfected. Progress of the infection was monitored by bioluminescence imaging. Imaging was carried out by intraperitoneal injection of 150 mg/kg D-luciferin. After 5 min, mice were anaesthetised with 2.5% (v/v) gaseous isofluorane in oxygen. The mice were transferred to the IVIS Illumina and imaged using LivingImage 4.3. software (PerkinElmer). Exposure times were determined automatically and varied between 1 s and 5 min depending on the radiance. After imaging, mice were allowed to recover and were transferred back to their cages. These studies were conducted in two separate experiments. The experiment using BALB/c mice (Fig. 4a) and that in which ISG65+/+ trypanosomes were used to infect C57BL/6J or C57BL/6J C3−/− mice (Fig. 4b) were conducted concurrently, and the two sets of mice were infected using the same trypanosome culture. The experiment in which C57BL/6J or C57BL/6J C3−/− mice were infected with ISG65−/− trypanosomes (Fig. 4c) was conducted subsequently and, although the same trypanosome cell line was used as above, should not be directly compared with the experiment presented in Fig. 4a, b.

### Reporting summary

Further information on research design is available in the Nature Research Reporting Summary linked to this article.

## Supplementary information

Supplementary information

Description of Additional Supplementary Information

supplementary data 1

supplementary data 2

Reporting Summary

## Data Availability

The *T. brucei* EATRO1125 ISG65 A to G gene sequences used in this study have been deposited in the European Nucleotide Archive under accession codes OU529038, OU529039, OU529040, OU529041, OU529042, OU529043 and OU529044 and are given, together with other sequences, in “supplementary data 1.xlsx” The raw reads from the genome sequencing used in this study have been deposited in the ArrayExpress database under accession code E-MTAB-10878. Coordinates and structure factors used in this study have been deposited in the Protein Data Bank under accession code 7PI6. Uncropped gels generated in this study are provided in “source data.xlsx”, while those relating to supplementary information are at the end of the Supplementary Information file. Raw SAXS data generated in this study is provided in “supplementary data 2.zip”. Source data are provided with this paper.

## Source data

Source Data

## References

[1] Sudarshi D (2014). Human African Trypanosomiasis presenting at least 29 years after infection—what can this teach us about the pathogenesis and control of this neglected tropical disease?. PLoS Negl. Trop. Dis..

[2] Schwede A, Macleod OJS, MacGregor P, Carrington M (2015). How does the VSG coat of bloodstream form African Trypanosomes interact with external proteins?. PLoS Pathog..

[3] Schwede A, Jones N, Engstler M, Carrington M (2011). The VSG C-terminal domain is inaccessible to antibodies on live trypanosomes. Mol. Biochem. Parasitol..

[4] Schwede A, Carrington M (2010). Bloodstream form trypanosome plasma membrane proteins: antigenic variation and invariant antigens. Parasitology.

[5] Ziegelbauer K, Rudenko G, Kieft R, Overath P (1995). Genomic organization of an invariant surface glycoprotein gene family of *Trypanosoma brucei*. Mol. Biochem. Parasitol..

[6] Ziegelbauer K, Multhaup G, Overath P (1992). Molecular characterization of 2 invariant surface glycoproteins specific for the blood-stream stage of T*rypanosoma brucei*. J. Biol. Chem..

[7] Jackson DG, Windle HJ, Voorheis HP (1993). The identification, purification, and characterization of 2 invariant surface glycoproteins located beneath the surface-coat barrier of blood-stream forms of Trypanosoma-Brucei. J. Biol. Chem..

[8] Ziegelbauer K, Overath P (1993). Organization of 2 invariant surface glycoproteins in the surface-coat of *Trypanosoma brucei*. Infect. Immun..

[9] Janssen BJC, Christodoulidou A, McCarthy A, Lambris JD, Gros P (2006). Structure of C3b reveals conformational changes that underlie complement activity. Nature.

[10] Schatz-Jakobsen JA, Pedersen DV, Andersen GR (2016). Structural insight into proteolytic activation and regulation of the complement system. Immunol. Rev..

[11] Menny A (2018). CryoEM reveals how the complement membrane attack complex ruptures lipid bilayers. Nat. Commun..

[12] Xue XG (2017). Regulator-dependent mechanisms of C3b processing by factor I allow differentiation of immune responses. Nat. Struct. Mol. Biol..

[13] Schmidt CQ, Lambris JD, Ricklin D (2016). Protection of host cells by complement regulators. Immunol. Rev..

[14] Forneris F (2016). Regulators of complement activity mediate inhibitory mechanisms through a common C3b-binding mode. EMBO J..

[15] Moore SR, Menon SS, Cortes C, Ferreira VP (2021). Hijacking factor H for complement immune evasion. Front. Immunol..

[16] Lambris JD, Ricklin D, Geisbrecht BV (2008). Complement evasion by human pathogens. Nat. Rev. Microbiol..

[17] Trevor CE (2019). Structure of the trypanosome transferrin receptor reveals mechanisms of ligand recognition and immune evasion. Nat. Microbiol..

[18] Salmon D (1994). A novel heterodimeric transferrin receptor encoded by a pair of VSG expression site-associated genes in *T. brucei*. Cell.

[19] Steverding D (1994). ESAG 6 and 7 products of *Trypanosoma brucei* form a transferrin binding protein complex. Eur. J. Cell Biol..

[20] Lane-Serff H, MacGregor P, Lowe ED, Carrington M, Higgins MK (2014). Structural basis for ligand and innate immunity factor uptake by the trypanosome haptoglobin-haemoglobin receptor. Elife.

[21] Vanhollebeke B (2008). A haptoglobin-hemoglobin receptor conveys innate immunity to Trypanosoma brucei in humans. Science.

[22] Macleod OJS (2020). A receptor for the complement regulator factor H increases transmission of trypanosomes to tsetse flies. Nat. Commun..

[23] Ricklin D, Reis ES, Mastellos DC, Gros P, Lambris JD (2016). Complement component C3-The “Swiss Army Knife” of innate immunity and host defense. Immunol. Rev..

[24] Berriman M (2005). The genome of the African trypanosome *Trypanosoma brucei*. Science.

[25] Muller LSM (2018). Genome organization and DNA accessibility control antigenic variation in trypanosomes. Nature.

[26] Chung WL, Carrington M, Field MC (2004). Cytoplasmic targeting signals in transmembrane invariant surface glycoproteins of trypanosomes. J. Biol. Chem..

[27] Rooijakkers SHM (2009). Structural and functional implications of the alternative complement pathway C3 convertase stabilized by a staphylococcal inhibitor. Nat. Immunol..

[28] Forneris F (2010). Structures of C3b in complex with factors B and D give insight into complement convertase formation. Science.

[29] Bajic G, Yatime L, Klos A, Andersen GR (2013). Human C3a and C3a desArg anaphylatoxins have conserved structures, in contrast to C5a and C5a desArg. Protein Sci..

[30] Janssen BJC (2005). Structures of complement component C3 provide insights into the function and evolution of immunity. Nature.

[31] De Crescenzo G, Grothe S, Lortie R, Debanne MT, O’Connor-McCourt M (2000). Real-time kinetic studies on the interaction of transforming growth factor alpha with the epidermal growth factor receptor extracellular domain reveal a conformational change model. Biochemistry.

[32] Hammel M (2007). A structural basis for complement inhibition by *Staphylococcus aureus*. Nat. Immunol..

[33] Burman JD (2008). Interaction of human complement with Sbi, a staphylococcal immunoglobulin-binding protein: indications of a novel mechanism of complement evasion by Staphylococcus aureus. J. Biol. Chem..

[34] Hammel M (2007). Characterization of Ehp, a secreted complement inhibitory protein from Staphylococcus aureus. J. Biol. Chem..

[35] Morgan HP (2011). Structural basis for engagement by complement factor H of C3b on a self surface. Nat. Struct. Mol. Biol..

[36] Wu J (2009). Structure of complement fragment C3b-factor H and implications for host protection by complement regulators. Nat. Immunol..

[37] Higgins MK (2013). Structure of the trypanosome haptoglobin-hemoglobin receptor and implications for nutrient uptake and innate immunity. Proc. Natl Acad. Sci. USA.

[38] Webb H (1997). The GPI-phospholipase C of *Trypanosoma brucei* is nonessential but influences parasitemia in mice. J. Cell Biol..

[39] Mugnier MR, Cross GA, Papavasiliou FN (2015). The in vivo dynamics of antigenic variation in *Trypanosoma brucei*. Science.

[40] Devine DV, Falk RJ, Balber AE (1986). Restriction of the alternative pathway of human-complement by intact *Trypanosoma Brucei Subsp Gambiense*. Infect. Immun..

[41] Liu EW, Otesile EB, Tabel H (1993). Immune lysis of *Trypanosoma congolense*—generation of a soluble covalent complex of variant surface glycoprotein and bovine complement component-C3b. Vet. Immunol. Immunopathol..

[42] Engstler M (2007). Hydrodynamic flow-mediated protein sorting on the cell surface of trypanosomes. Cell.

[43] Sullivan L, Wall SJ, Carrington M, Ferguson MAJ (2013). Proteomic selection of immunodiagnostic antigens for human African trypanosomiasis and generation of a prototype lateral flow immunodiagnostic device. PLoS Negl. Trop. Dis..

[44] Dhalia R (2006). The two eIF4A helicases in *Trypanosoma brucei* are functionally distinct. Nucleic Acids Res..

[45] Sunter J, Wickstead B, Gull K, Carrington M (2012). A new generation of T7 RNA polymerase-independent inducible expression plasmids for *Trypanosoma brucei*. PLoS ONE.

[46] Trudgian DC (2011). Comparative evaluation of label-free SINQ normalized spectral index quantitation in the central proteomics facilities pipeline. Proteomics.

[47] Winter G (2018). DIALS: implementation and evaluation of a new integration package. Acta Crystallogr. Sect. D. Struct. Biol..

[48] Winn MD (2011). Overview of the CCP4 suite and current developments. Acta Crystallogr. Sect. D. Struct. Biol..

[49] Potterton L (2018). CCP4i2: the new graphical user interface to the CCP4 program suite. Acta Crystallogr. Sect. D. Struct. Biol..

[50] Mccoy AJ (2007). Phaser crystallographic software. J. Appl. Crystallogr..

[51] Nagar B, Jones RG, Diefenbach RJ, Isenman DE, Rini JM (1998). X-ray crystal structure of C3d: A C3 fragment and ligand for complement receptor 2. Science.

[52] Emsley P, Lohkamp B, Scott WG, Cowtan K (2010). Features and development of Coot. Acta Crystallogr. Sect. D. Biol. Crystallogr..

[53] Bricogne, G. et al. *BUSTER version 2.10.4* (Global Phasing Ltd., 2017).

[54] Cowieson NP (2020). Beamline B21: high-throughput small-angle X-ray scattering at diamond light Source. J. Synchrotron Radiat..

[55] Manalastas-Cantos K (2021). ATSAS 3.0: expanded functionality and new tools for small-angle scattering data analysis. J. Appl. Crystallogr..

[56] Grant TD (2018). Ab initio electron density determination directly from solution scattering data. Nat. Methods.

[57] Wiedemann C, Bellstedt P, Gorlach M (2013). CAPITO-a web server-based analysis and plotting tool for circular dichroism data. Bioinformatics.

[58] Van Meirvenne N, Janssens PG, Magnus E (1975). Antigenic variation in syringe passaged populations of *Trypanosoma (Trypanozoon) brucei*. 1. Rationalization of the experimental approach. Ann. Soc. Belg. Med Trop..

[59] Costa FC (2018). Expanding the toolbox for *Trypanosoma cruzi*: a parasite line incorporating a bioluminescence-fluorescence dual reporter and streamlined CRISPR/Cas9 functionality for rapid in vivo localisation and phenotyping. PLoS Negl. Trop. Dis..

[60] Beneke T (2017). A CRISPR Cas9 high-throughput genome editing toolkit for kinetoplastids. R. Soc. Open Sci..

[61] Bolger AM, Lohse M, Usadel B (2014). Trimmomatic: a flexible trimmer for Illumina sequence data. Bioinformatics.

[62] Langmead B, Salzberg SL (2012). Fast gapped-read alignment with Bowtie 2. Nat. Methods.

[63] Afgan E (2018). The Galaxy platform for accessible, reproducible and collaborative biomedical analyses: 2018 update. Nucleic Acids Res..

[64] Carver T, Harris SR, Berriman M, Parkhill J, McQuillan JA (2012). Artemis: an integrated platform for visualization and analysis of high-throughput sequence-based experimental data. Bioinformatics.

[65] Wang S, Ma JZ, Xu JB (2016). AUCpreD: proteome-level protein disorder prediction by AUC-maximized deep convolutional neural fields. Bioinformatics.

[66] Pettersen EF (2004). UCSF chimera—a visualization system for exploratory research and analysis. J. Comput. Chem..

[67] Goddard TD, Huang CC, Ferrin TE (2007). Visualizing density maps with UCSF Chimera. J. Struct. Biol..

