# Supplementary material for: Invariant surface glycoprotein 65 of Trypanosoma brucei is a complement C3 receptor
Source: Nat Commun. 2022 Aug 29;13:5085. doi: 10.1038/s41467-022-32728-9 (PMC9424271; doi:10.1038/s41467-022-32728-9)

Supplementary Information

Invariant surface glycoprotein 65 of *Trypanosoma brucei* is a complement C3 receptor

Supplementary Data Figures and Tables

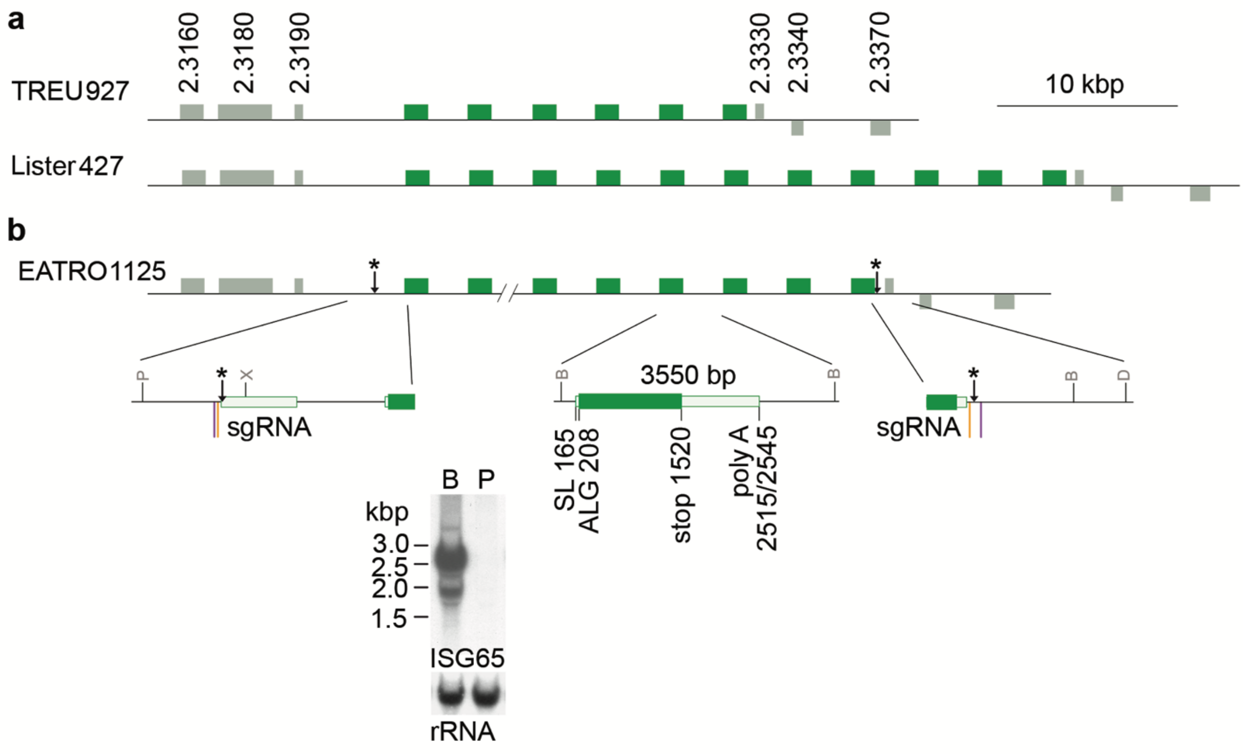

Supplementary Fig. 1: ISG65 loci and strategies to delete the locus

a. The ISG65 loci from the mosaic haploid genome sequences of *T. brucei* TREU927[24] and Lister 427[25]. ISG65 open reading frames are shown in green and others in grey. Boxes above the line indicate transcription from left to right and below the line from right to left. Open reading frames annotated in the genome but present within the 3'UTR of *ISG65* mRNA have been removed.
b. The upper panel shows a map of the ISG65 locus from *T. brucei* EATRO1125 derived from two cosmid clones. The break in the map represents the ends of the insert in the two cosmids and the number of missing ISG65 genes was not determined. Genes are indicated as in (A). The * symbols mark the ends of tandemly repeated ISG65 sequences. The location of the sgRNA is shown as an orange line and the 80 bp flanking sequence for insertion of the selectable marker cassette as a purple line. The lower panel shows a northern blot of total RNA from bloodstream (B) and procyclic (P) forms to show the size of the *ISG65* mRNA, n=1.

a

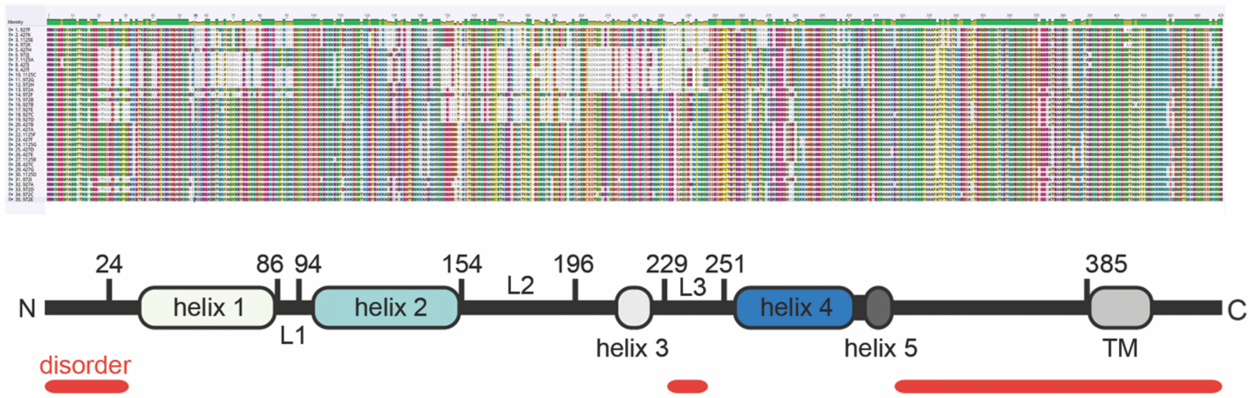

b

	1125A	1125B	1125C	1125D	1125E	1125F	1125G
1125A		0.36	0.07	0.37	0.32	0.35	0.36
1125B			0.38	0.03	0.23	0.01	0.01
1125C				0.39	0.32	0.38	0.38
1125D					0.22	0.03	0.03
1125E						0.22	0.22
1125F							0.01
1125G							

c

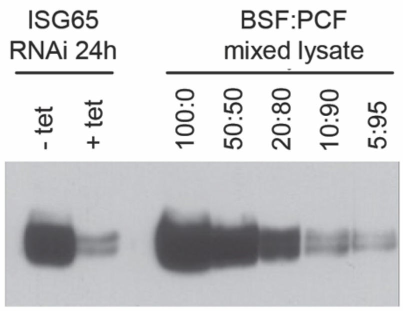

Supplementary Fig. 2: characterisation of ISG65 variation and knock-down

a. Alignment of polypeptide sequences of ISG65 from *T. brucei* EATRO1125 determined in this study and from the genome sequences of *T. brucei* TREU927 and Lister427 produced using Clustal Omega and using default settings in Geneious Prime 2021.2.2 (https://www.geneious.com). A larger scale alignment can be seen in Supplementary Fig. 8 and sequences are provided in “supplementary data 1.xlsx”. Below the alignment is a schematic of the structural features of ISG65, aligned against the sequence alignment. The red bars are regions predicted to be disordered as in Fig. 3. **b.** Difference matrix to show sequence identity between the ISG65 proteins from *T. brucei* EATRO1125 was prepared after a global alignment and Blosum 62 cost matrix via Geneious Prime 2021.2.2. The difference between ISG65A and G is highlighted in yellow. **c.** Western blot showing tetracycline-inducible depletion of ISG65 (n=1). *T. brucei* Lister427 pSPR2 p295 was grown in culture and RNAi induced for 24 h prior to analysing ISG65 knock down by western blotting. The same number of cell equivalents were loaded in each track. Comparison with standard curve of bloodstream form cells indicated there was ~90% depletion of ISG65 protein. These cells were used for immunofluorescence in Fig. 1b.

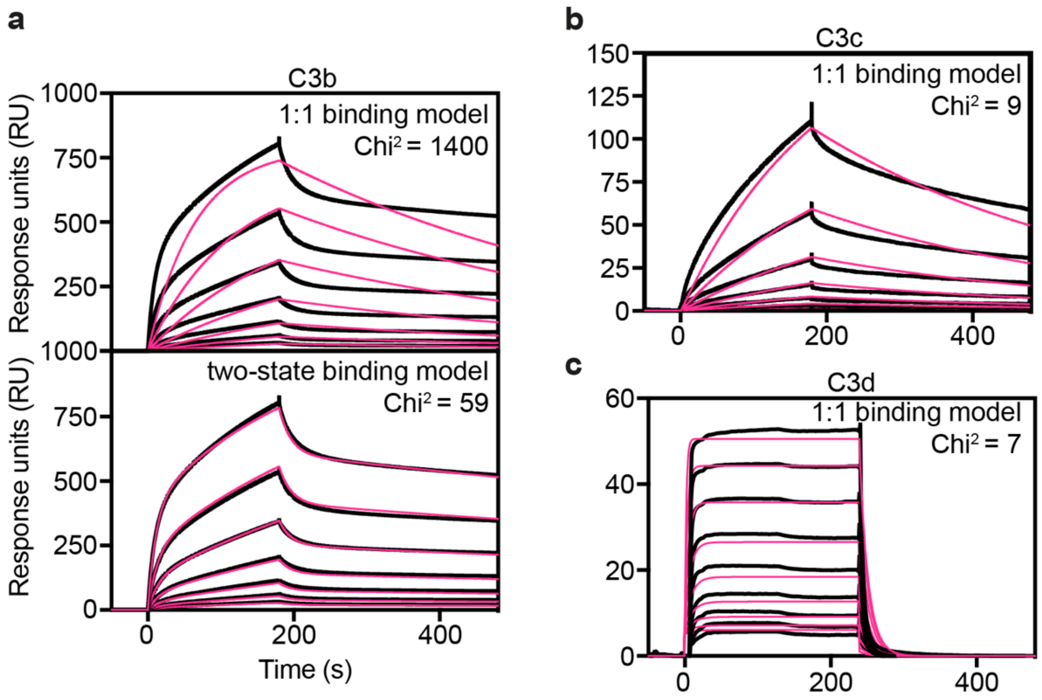

Supplementary Fig. 3: Surface plasmon resonance analysis of binding of C3 fragments to ISG65

ISG65G was immobilised on an SPR chip surface for assessment of binding of: **a.** C3b (a two-folding dilution series from a starting concentration of 0.5 μM); **b.** C3c (a two-folding dilution series from a starting concentration of 0.5 μM) and **c.** C3d (a two-folding dilution series from a starting concentration of 4 μM). Data was fitted to either a 1:1 binding model or a two-state binding model, as indicated, with the chi^2 for kinetic fitting reported, n=3.

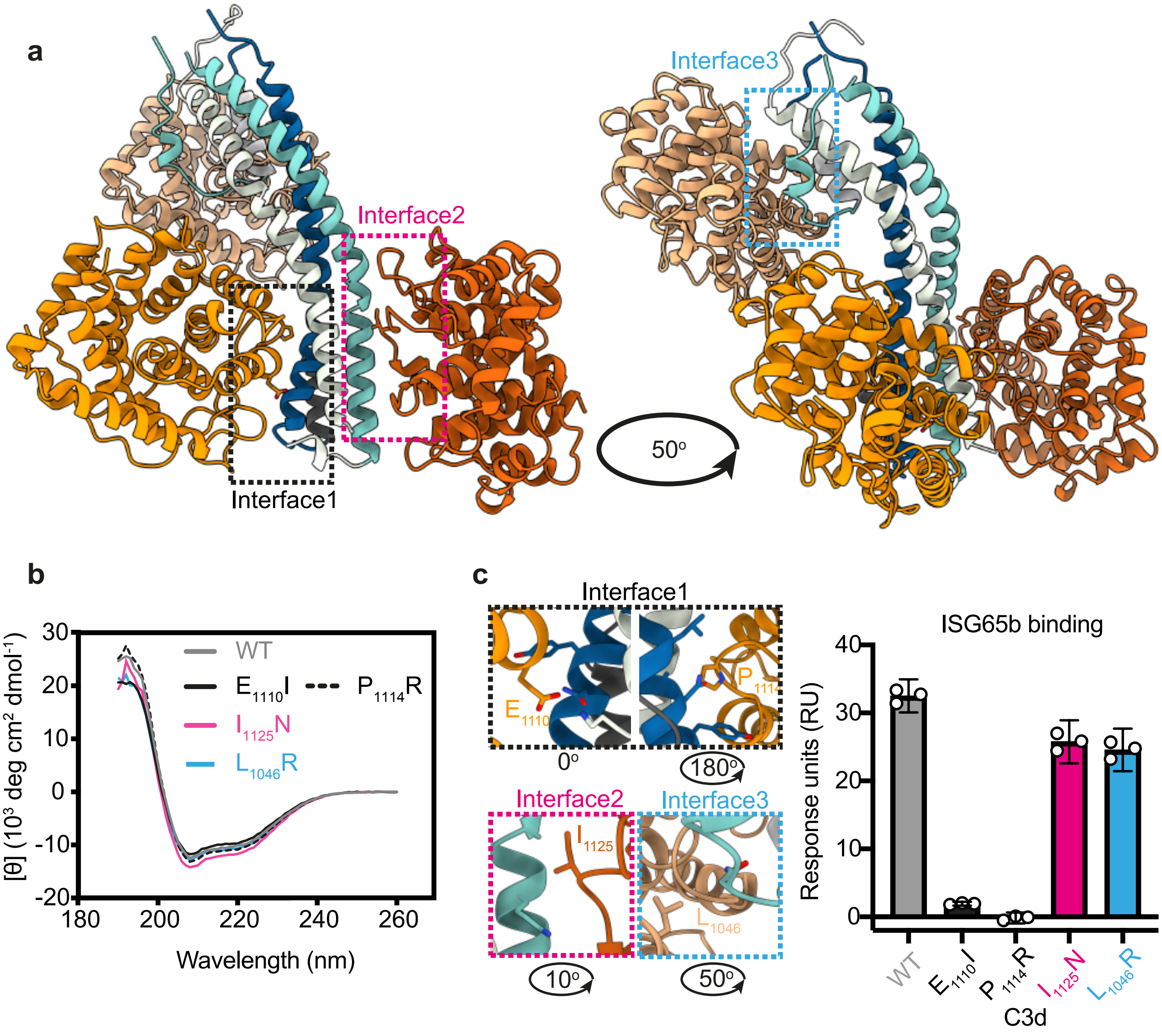

Supplementary Fig. 4: Determination of the ISG65-C3d interface

a. The crystal structure of ISG65-C3d revealed three possible interfaces between ISG65 and C3d. These were interface 1 (black box, orange C3d), interface 2 (pink box, dark orange C3d), and interface 3 (blue box, light orange C3d). **b.** Circular dichroism spectra of C3d and mutants designed to disrupt interface 1, 2, and 3. **c.** The left-hand panel shows close up views of the interfaces, coloured as **a.** and highlighting the mutations made to disrupt interfaces 1, 2, and 3. The right-hand panel shows the average binding of C3d and mutants to ISG65 (n = 3 technical replicates, with error bars denoting standard deviation) as measured by surface plasmon resonance analysis.

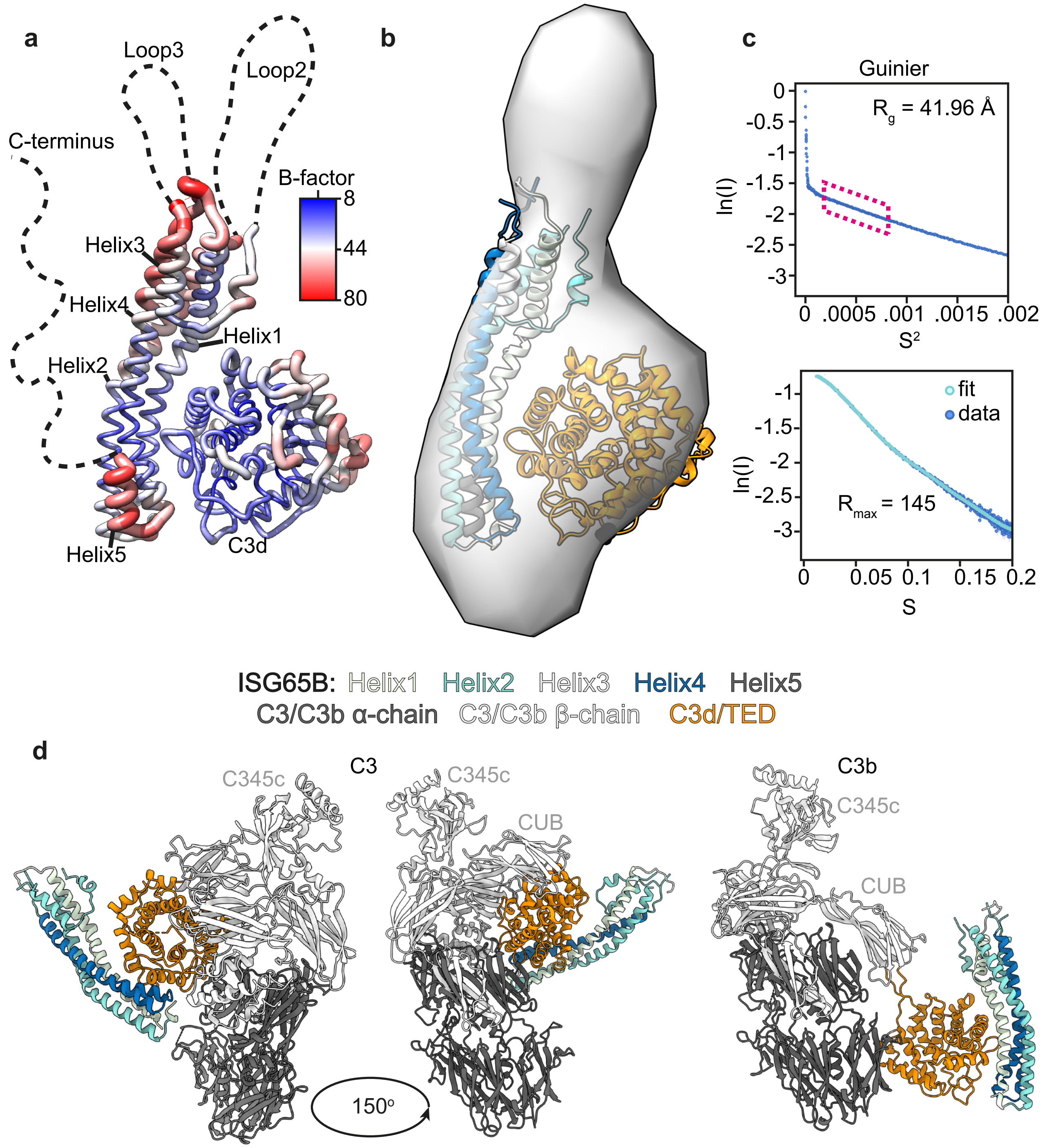

Supplementary Fig. 5: Analysis of the ISG65-C3d complex

a. The ISG65-C3d structure is shown as 'putty' with the width of the coil and the colour determined by B-factor. Dotted lines indicate the unresolved loops 2 and 3, and the C-terminal linker. **b.** The ISG65-C3d structure rigid body-fitted into the corresponding SAXS density. Initial fitting was performed using the Chimera fit_in_map tool[67] at a resolution of 15 Å, and then manually adjusted to account for the large density with no corresponding structure. This density is most likely attributable to loop L2. **c.** The top panel shows a Guinier plot of SAXS data, with a pink box showing the linear Guinier region used for fitting, and the resultant estimate of radius of gyration (R_g). The bottom panel shows SAXS data with corresponding fit and R_{max} value estimate, used for *ab initio* reconstruction of a 3D volume. Raw scattering curves are provided in "supplementary data 2". **d.** Models of ISG65 bound to complement C3 and C3b. The ISG65-C3d structure was docked onto structures of C3 (PDB: 2A73)[30] and C3b (PDB: 2I07)[9], with the light chain in light grey and the heavy chain in dark grey.

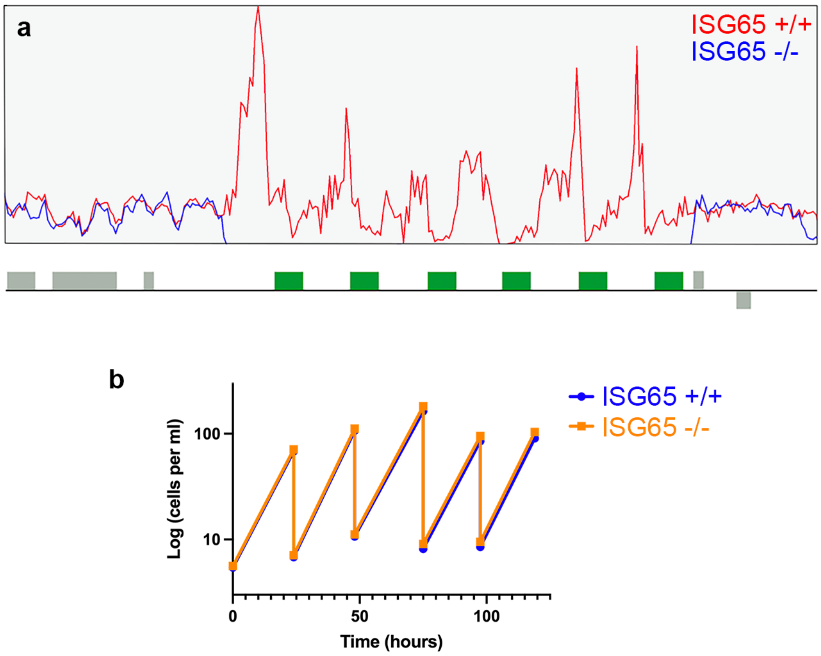

Supplementary Fig. 6: confirmation of ISG65 knockout in cell lines and assessment of its growth in culture

a. Mapping of sequencing reads from parental (red) and ISG65 knock-out (blue) cell lines onto the *T. brucei* TREU927 reference genome. ISG65 open reading frames are shown in green and others in grey. Boxes above the line indicate transcription from left to right and below the line from right to left. **b.** Growth in culture of *T. brucei* EATRO1125 ISG65+/+ (blue) and ISG65-/- (orange). Cells were passaged each day for 5 days.

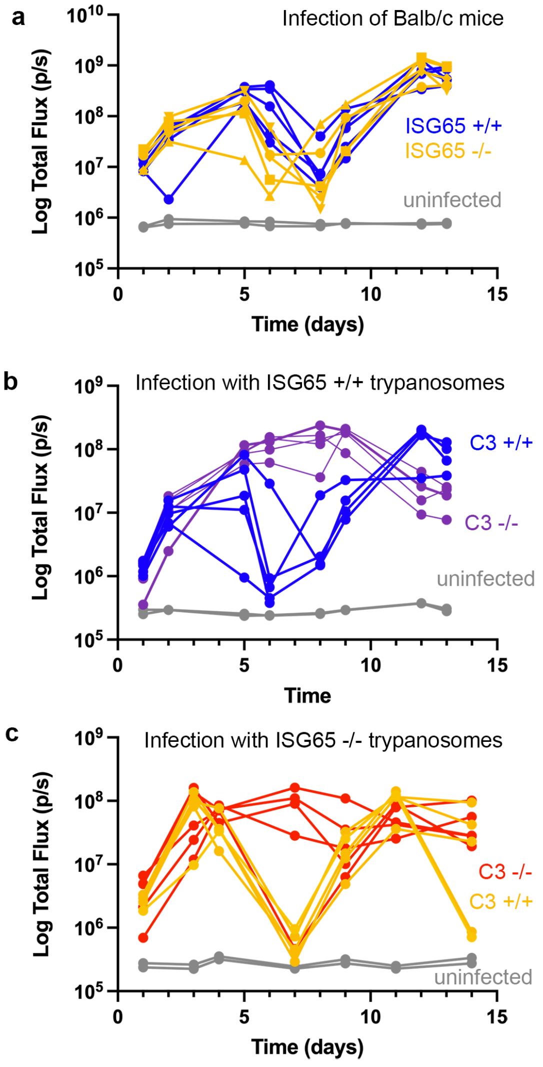

Supplementary Fig. 7: Assessment of the effect of ISG65 and C3 knock-out on trypanosome growth in mice

a. The effect of ISG65 knock-out on trypanosome infections in Balb/c mice. Five mice were infected with bioluminescent ISG65+/+ (blue, n=5 mice) or ISG65-/- (orange, n=5 mice) *T. brucei* cell lines. Two uninfected mice were used as controls for basal bioluminescence (grey, n=2 mice). Trypanosome burden was measured by imaging bioluminescence over time. **b.** The effect of C3 knock out on trypanosome infections in C57BL/6 mice. Bioluminescent ISG65+/+ *T. brucei* were used to infect five C57BL/6 mice (blue, n=5 mice) and five C57BL/6 mice lacking C3 (purple, n=5 mice). Two uninfected mice were used as controls for basal bioluminescence (grey, n=2 mice). Trypanosome burden was measured by imaging bioluminescence over time. **c.** The effect of C3 knock out on ISG65-/- trypanosome infections in C57BL/6 mice. Bioluminescent ISG65-/- *T. brucei* were used to infect five C57BL/6 mice (orange, n=5 mice) and five C57BL/6 mice lacking C3 (red, n=5 mice). These two infections were not done in parallel. Two uninfected mice were used as controls for basal bioluminescence (grey, n=2 mice). Trypanosome burden was measured by imaging bioluminescence over time. The data presented in Fig. 4a and 4b. were conducted concurrently, while that presented in Fig. 4c was conducted subsequently and should not be directly compared with the data in Fig 4a and 4b.

Supplementary Fig. 8: Alignment of ISG65 proteins analysed in this study

```
                   10        20        30        40        50        60        70        80
1125E     MMKYLLVFAIITTRISVLLVIGSEDNRVPGDKKLTKEGAAALCKMKHLADKVAEKGAEDLKKKTKNFAGFIEFEQEKVDN   80
427K      MMKYLLVFAIITTRISVLLVIGSEDNRVPGDKKLTKEGAAALCKMKHLADKVAEKGAEDLKKKTKNFAGFIEFEQEKVDN   80
927F      MMKYLLVFAIITTRIPVLLVIGSEDNRVPGDKKLTKEGAAALCKMKHLADKVAEKGAEDLKKKTQNFAGFIEFEQEKVDN   80
427H      MMKYLLVFAIITTRISVLLATNGGDKHVKANKKLTKEGAAALCKMKHLADKVAEERSPELKDRTQNFAGYIEFELYRIDY   80
1125C     MMKYLLVFAIITTRISVLLATNGGDKHVKANKKLDQEGANLLCKMKHLADKVANKGGEDLKKKTKGFEDDVLLEVERVNN   80
427I      MMKYLLVFAIITTRISVLLATNGGDKHVKANKKLDQEGANLLCKMKHLADKVANKGGEDLKKKTKGFEDDVLLEVERVNN   80
1125A     MMKYLIVFAIITTRISVLLATNGGDKHVKANKKLDQEGANLLCKMKHLADKVANKGGEDLKKKTKGFEDDVLLEVERVNN   80
427J      MMKYLIVFAIITTRISVLLATNGGDKHVKANKKLDQEGANLLCKMKHLADKVANKGGEDLKKKTKGFEDDVLLEVERVNN   80
927E      MMKYLLVFAIIATRIPVLLATNGGDKHVKANKKLTKEGAAALCKMKHLADKVAEERSQELKDRTQIFAGYIEFELYRIDY   80
927B      MMKYLLVFAIIATRISVLLATNGGDKHVKANKKLTKEGAAALCKMKHLADKVAEERSQELKDRTQNFAGYIEFELYRIDY   80
927C      MMKYLLVFAIIATRIPVLLATNGGDKHVKANKKLTKEGAAALCKMKHLADKVAEERSQELKDRTQNFAGYIEFELYRIDY   80
927D      MMKYLLVFAIIATRIPVLLATNGGDKHVKANKKLTKEGAAALCKMKHLADKVAEERSQELKDRTQNFAGYIEFELYRIDY   80
927A      MMKYLLVFAIIATRIPALLVIGSEDNRVPGDKKLTKEGAAALCKMKHLADKVAEERSQELKDRTQNFAGYIEFELYRIDY   80
427C      MMKYLLVFAIITTRISVLLVIGSEDNRVPGDKNLTKEGAAALCKMKHLADKVAEKRSQELKDRTQNFAGYIEFELYRIDY   80
1125D     MMKYLLVFAIITTRISVLLVIGSEDNRVPGDKNLTKEGAAALCKMKHLADKVAEKRSQELKDRTQNFAGYIEFELYRIDY   80
427G      MMKYLLVFAIITTRISVLLVIGSEDNRVPGDKNLTKEGAAALCKMKHLADKVAEKRSQELKDRTQNFAGYIEFELYRIDY   80
427B      MMKYLLVFAIITTRISVLLVIGSEDNRVPGDKNLTKEGAAALCKMKHLADKVAEKRSQELKDRTQNFAGYIEFELYRIDY   80
427D      MMKYLLVFAIITTRISVLLVIGSEDNRVPGDKNLTKEGAAALCKMKHLADKVAEKRSQELKDRTQNFAGYIEFELYRIDY   80
427E      MMKYLLVFAIITTRISVLLVIGSEDNRVPGDKNLTKEGAAALCKMKHLADKVAEKRSQELKDRTQNFAGYIEFELYRIDY   80
427A      MMKYLLVFAIITTRISVLLVIGSEDNRVPGDKNLTKEGAAALCKMKHLADKVAEKRSQELKDRTQNFAGYIEFELYRIDY   80
1125F     MMKYLLVFAIITTRISVLLVIGSEDNRVPGDKNLTKEGAAALCKMKHLADKVAEKRSQELKDRTQNFAGYIEFELYRIDY   80
1125B     MMKYLLVFAIITTRISVLLVIGSEDNRVPGDKNLTKEGAAALCKMKHLADKVAEKRSQELKDRTQNFAGYIEFELYRIDY   80
1125G     MMKYLLVFAIITTRISVLLVIGSEDNRVPGDKNLTKEGAAALCKMKHLADKVAEKRSQELKDRTQNFAGYIEFELYRIDY   80
427F      MMKYLLVFAIITTRISVLLVIGSEDNRVPGDKNLTKEGAAALCKMKHLADKVAEKRSQELKDRTQNFAGYIEFELYRIDY   80

                   90       100       110       120       130       140       150       160
1125E     WLEKLRNRKQYSDGYAKLSDSDVEKVKEIFDKAKDGITKQIPEAEKEAREAERLYDEVKKAAQDARGQDLDDDTAKSTGL  160
427K      WLEKLRNRKQYSDGYAKLSDSDVEKVKEIFDKAKDGITKQIPEAEKEAREAERLYDEVKKAAQDARGQDLDDDTAKSTGL  160
927F      WLEKLRNRKQYSDGYAKLSDSDVEKVKEIFDKAKDGITKQLPEAEKEAREAERLYDEVKKAAQDARGQDLDDDTAKSTGL  160
427H      WLEKLNGPKGRKDGYAKLSDSDIEKVKEIFNKAKDGITKQLPEAKKAADDAKKLYDEVKKAAEDARGVKVSDE-TNSSGL  159
1125C     WLEKLGDRKQYNDGYAKLSDSDIEKVKGIFTKAKDGITKQLPEAKKAADDAKKLYDEVKKAAEDARGVKVSDE-TNSSGL  159
427I      WLEKLGDRKQYNDGYAKLSDSDIEKVKGIFTKAKDGITKQLPEAKKAADDAKKLYDEVKKAAEDARGVKVSDE-TNSSGL  159
1125A     WLEKLNGPKGRKDGYAKLSDSDIEKVKGIFTKAKDGITKQLPEAKKAADDAKKLYDEVKKAAEHARGVKVSDE-TNSSGL  159
427J      WLEKLNGPKGRKDGYAKLSDSDIEKVKGIFTKAKDGITKQLPEAKKAADDAKKLYDEVKKAAEHARGVKVSDE-TNSSGL  159
927E      WLEKLNGPKGRKDGYAKLSDSDIEKVKEIFNKAKDGIAKQLPEAKKAGEEAEKLHTEVKKAAANARGVRLSEG-TNSSGL  159
927B      WLEKLNGPKGRKDGYAKLSDSDIEKVKEIFNKAKDGITKQLPEAKKAGEEAEKLHTEVKKAAKNARGVRLSEG-TNSSGL  159
927C      WLEKLNGPKGRKDGYAKLSDSDIEKVKEIFNKAKDGIAKQLPEAKKAGEEAEKLHTEVKKAAKNARGVRLSEG-TNSSGL  159
927D      WLEKLNGPKGRKDGYAKLSDSDIEKVKEIFNKAKDGIAKQLPEAKKAGEEAEKLHTEVKKAAKNARGVRLSEG-TNSSGL  159
927A      WLEKLNGPKGRKDGYAKLSDSDIEKVKEIFNKAKDGIAKQLPEAKKAGEEAEKLHTEVKKAAANARGQDLDEYRQKSTGL  160
427C      WLEKLNGPKGRKDGYAKLSDSDIEKVKEIFDKAKDGIAKQLPEAKKAGEDAEKLHTEVKEAAANARGQDLDDHKQKSTGL  160
1125D     WLEKLNGPKGRKDGYAKLSDSDIEKVKEIFDKAKDGIAKQLPEAKKAGEDAEKLHTEVKEAAANARGQDLDDHKQKSTGL  160
427G      WLEKLNGPKGRKDGYAKLSDSDIEKVKEIFDKAKDGIAKQLPEAKKAGEDAEKLHTEVKEAAANARGQDLDDHKQKSTGL  160
427B      WLEKLNGPKGRKDGYAKLSDSDIEKVKEIFDKAKDGIAKQLPEAKKAGEDAEKLHTEVKEAAANARGQDLDDHKQKSTGL  160
427D      WLEKLNGPKGRKDGYAKLSDSDIEKVKEIFDKAKDGIAKQLPEAKKAGEDAEKLHTEVKEAAANARGQDLDDHKQKSTGL  160
427E      WLEKLNGPKGRKDGYAKLSDSDIEKVKEIFDKAKDGIAKQLPEAKKAGEDAEKLHTEVKEAAANARGQDLDDHKQKSTGL  160
427A      WLEKLNGPKGRKDGYAKLSDSDIEKVKEIFDKAKDGIAKQLPEAKKAGEDAEKLHTEVKEAAANARGQDLDDHKQKSTGL  160
1125F     WLEKLNGPKGRKDGYAKLSDSDIEKVKEIFDKAKDGIAKQLPEAKKAGEDAEKLHTEVKEAAANARGQDLDDHKQKSTGL  160
1125B     WLEKLNGPKGRKDGYAKLSDSDIEKVKKIFDKAKDGIAKQLPEAKKAGEDAEKLHTEVKEAAANARGQDLDDHKQKSTGL  160
1125G     WLEKLNGPKGRKDGYAKLSDSDIEKVKEIFDKAKDGIAKQLPEAKKAGEDAEKLHTEVKEAAANARGQDLDDHKQKSTGL  160
427F      WLEKLNGPKGRKDGYAKLSDSDIEKVKEIFDKAKDGIAKQLPEAKKAGEDAEKLHTEVKEAAANARGQDLDDHKQKSTGL  160

                  170       180       190       200       210       220       230       240
Consensus YRVLNWYCITKEENHNATPNCD-GIQFRNHYLSVN-RSAIDCSSTGYEENYDWSANALQVALNSWENVKPKKLE-SAGSD  237
1125E     YRVLNWYCITKDNNKDITHNCDDGIKFRDHYLSVK-RSAIDCSSTGYKEDYDWSANALQSALNDWENVKPKEVKPESGGN  239
427K      YRVLNWYCITKDNNKDITHNCDDGIKFRDHYLSVK-RSAIDCSSTGYKEDYDWSANALQSALNDWENVKPKEVKPESGGN  239
927F      YRVLNWYCITKDNNKDITHNCDDGIKFRDHYLSVN-RSAIDCSSTGYKEDYDWSANALQSALNDWENVKPKEVKPESGGN  239
427H      YRILDWYCFKEGENAGQSHNCE-NVGFSVHHGTHKRRNVIDCGDEKANKYGDASSKTLEDTLKEWNVKKPSAET-NNNGN  237
1125C     YRILDWYCFKEGENAGQSHNCE-NVGFSVHHGTHKRRNVIDCGDEKANKYGDASSKTLEDTLKEWNVKKPSAET-NNNGN  237
427I      YRILDWYCFKEGENAGQSHNCE-NVGFSVHHGTHKRRNVIDCGDEKANKYGDASSKTLEDTLKEWNVKKPSAET-NNNGN  237
1125A     YRILDWYCFKEGENAGKSDNCD-GVKFSEHHGTHRRRNVIDCGDEKANKYGDASSKTLEDTLKQWESKKPQAAG-ASGGN  237
427J      YRILDWYCFKEGENAGKSDNCD-GVKFSEHHGTHRRRNVIDCGDEKANKYGDASSKTLEDTLKQWESKKPQAAG-ASGGN  237
927E      YRILDWYCFKEGENAGKSDNCD-GVKFSVHYETHRRRNVIDCSSTGYEENYDWSANALQVALNSWENVKPKKLE-SAGSD  237
927B      YRILDWYCFKEGENAGKSDNCD-GVKFSVHYETHRRRNVIDCSSTGYEENYDWSANALQVALNSWENVKPKKLE-SAGSD  237
927C      YRILDWYCFKEGENAGKSDNCD-GVKFSVHYETHRRRNVIDCSSTGYEENYDWSANALQVALNSWENVKPKKVE-SAGSD  237
927D      YRILDWYCFKEGENAGKSDNCD-GVKFSVHYETHRRRNVIDCSSTGYEENYDWSANALQVALNSWENVKPKKVE-SAGSD  237
927A      YRVLNWYCITKEERHNATPNCD-GIQFRKHYLSVN-RSAIDCSSTGYEENYDWSANALQVALNSWENVKPKKLE-SAGSD  237
427C      YRVLNWYCITKEESHNATPNCD-GIQFRNHYLSVN-RSAIDCSSTGYEENYDWSANALQVALNSWENVKPKKLE-SAGSD  237
1125D     YRVLNWYCITKEESHNATPNCD-GIQFRNHYLSVN-RSAIDCSSTGYEENYDWSANALQVALNSWENVKPKKLE-SAGSD  237
427G      YRVLNWYCITKEESHNATPNCD-GIQFRNHYLSVN-RSAIDCSSTGYEENYDWSANALQVALNSWENVKPKKLE-SAGSD  237
427B      YRVLNWYCITKEESHNATPNCD-GIQFRNHYLSVN-RSAIDCSSTGYEENYDWSANALQVALNSWENVKPKKLE-SAGSD  237
427D      YRVLNWYCITKEESHNATPNCD-GIQFRNHYLSVN-RSAIDCSSTGYEENYDWSANALQVALNSWENVKPKKLE-SAGSD  237
427E      YRVLNWYCITKEESHNATPNCD-GIQFRNHYLSVN-RSAIDCSSTGYEENYDWSANALQVALNSWENVKPKKLE-SAGSD  237
427A      YRVLNWYCITKEESHNATPNCD-GIQFRNHYLSVN-RSAIDCSSTGYEENYDWSANALQVALNSWENVKPKKLE-SAGSD  237
1125F     YRVLNWYCITKEESHNATPNCD-GIQFRNHYLSVN-RSAIDCSSTGYEENYDWSANALQVALNSWENVKPKKLE-SAGSD  237
1125B     YRVLNWYCITKEESHNATPNCD-GIQFRNHYLSVN-RSAIDCSSTGYEENYDWSANALQVALNSWENVKPKKLE-SAGSD  237
1125G     YRVLNWYCITKEESHNATPNCD-GIQFRNHYLSVN-RSAIDCSSTGYEENYDWSANALQVALNSWENVKPKKLE-SAGSD  237
427F      YRVLNWYCITKEESHNATPNCD-GIQFRNHYLSVN-RSAIDCSSTGYEENYDWSANALQVALNSWENVKPKKLE-SAGSD  237
```

```
                      250         260       270       280       290       300       310       320
1125E     DVCKATASSESHPCTMTEEWQTHYKDSILKLKELEDAHKRGKAAHDAMLGYANTAHATNRKVEQEKPLAEVIAAAKDAGK  319
427K      DVCKATASSESHPCTMTEEWQTHYKDSILKLKELEDAHKRGKAAHDAMLGYANTAHATNRKVEQEKPLAEVIAAAKDAGK  319
927F      DVCKKDESSESHPCTMTGGWQTHYKDSILKLKELEDAHKRGKAAHDAMLGYANTAHAANTKVEQEKPLAEVITAAKDAGK  319
427H      DMCKNGQSSESHPCTMTEEWQTHYKETVKKLRELEGAHERGKKAHDAMLGYANTAHATNRKVEQGKPLTEVIAAAKEAGK  317
1125C     DMCKNGQSSESHPCTMTEEWQTHYKETVKKLRELEGAHERGKKAHDAMLGYANTAHATNRKVEQGKPLTEVIAAAKEAGK  317
427I      DMCKNGQSSESHPCTMTEEWQTHYKETVEKLRELEGAHERGKKAHDAMLGYANTAHATNRKVEQGKPLTEVIAAAKEAGK  317
1125A     DVCKATASSESHPCTMTEEWQTPYKETVKKLKELEGAHERGKKAHDAMLGYANTAHATNRKVEQGKPLTEVIAAAKEAGK  317
427J      DVCKATASSESHPCTMTEEWQTPYKETVKKLKELEGAHERGKKAHDAMLGYANTAHATNRKVEQGKPLTEVIAAAKEAGK  317
927E      MNCNIGQSSESHPCTMTEEWQTPYKETVEKLRELEDAYQRGKKAHDAMLGYANTAYAVNTKVEQEKPLTEVIAAAKEAGK  317
927B      MNCNIGQSSESHPCTMTEEWQTPYKETVEKLRELEDAYQRGKKAHDAMLGYANTAYAVNTKVEQEKPLTEVIAAAKEAGK  317
927C      MNCNIGQSSESHPCTMTEEWQTPYKETVEKLRELEDAYQRGKKAHDAMLGYANTAYAVNTKVEQEKPLTEVIAAAKDAGK  317
927D      MNCNIGQSSESHPCTMTEEWQTPYKETVEKLRELEDAYQRGKKAHDAMLGYANTAYAVNTKVEQEKPLTEVIAAAKEAGK  317
927A      MNCNIGQSSESHPCTMTEEWQTPYKETVEKLRELEDAYQRGKKAHDAMLGYANTAYAVNTKVEQEKPLTEVIAAAKDAGK  317
427C      ENCNIGQSSESHPCTMTEEWQTHYKETVKKLRELEDAYQRGKKAHDAMLGYANTAYAVNTKVEQEKPLAEVIAAAKEAGK  317
1125D     ENCNIGQSSESHPCTMTEEWQTHYKETVKKLRELEDAYQRGKKAHDAMLGYANTAYAVNTKVEQEKPLAEVIAAAKEAGK  317
427G      ENCNIGQSSESHPCTMTEEWQTHYKETVKKLRELEDAYQRGKKAHDAMLGYANTAYAVNTKVEQEKPLAEVIAAAKEAGK  317
427B      KNCNIGQSSESHPCTMTEEWQTHYKETVKKLKELEGAHEKGKKAHDAMLGYANTAYAVNTKVEQEKPLAEVIAAAKEAGK  317
427D      ENCNIGQSSESHPCTMTEEWQTHYKETVKKLKELEGAHEKGKRAHDAMLGYANTAYAVNTKVEQEKPLAEVIAAANEAGK  317
427E      ENCNIGQSSESHPCTMTEEWQTHYKETVKKLKELEGAHEKGKRAHDAMLGYANTAYAVNTKVEQEKPLAEVIAAANEAGK  317
427A      KNCNIGQSSESHPCTMTEEWQTHYKETVKKLKELEGAHEKGKKAHDAMLGYANTAYAVNTKVEQEKPLAEVIAAAKEAGK  317
1125F     KNCNIGQSSESHPCTMTEEWQTHYKETVKKLKELEGAHEKGKKAHDAMLGYANTAYAVNTKVEQEKPLAEVIAAAKEAGK  317
1125B     ENCNIGQSSESHPCTMTEEWQTHYKETVKKLKELEGAHEKGKRAHDAMLGYANTAYAVNTKVEQEKPLAEVIAAAKEAGK  317
1125G     ENCNIGQSSESHPCTMTEEWQTHYKETVKKLKELEGAHEKGRRAHDAMLGYANTAYAVNTKVEQEKPLAEVIAAAKEAGK  317
427F      ENCNIGQSSESHPCTMTEEWQTHYKETVKKLKELEGAHEKGRRAHDAMLGYANTAYAVNTKVEQEKPLAEVIAAAKEAGK  317

                      330         340       350       360       370       380       390       400
1125E     KGAKIIIPAAAPATSTDSTKSEDSAPTEHVDRGIATNETQVEVGIDADFDSLLEATEAAEVKSRHQRTAMIILAVLVPAI  399
427K      KGAKIIIPAAAPATSTDSTKSEDSAPTEHVDRGIATNETQVEVGIDADFDSLLEATEAAEVKSRHQRTAMIILAVLVPAI  399
927F      KGAKIIIPAAAPATSTDSTKSEDSAPAEHVDRGIATNETQVEVGIDADFDSLLEAAEAAEVKSRHQRTAMIILAVLVPAI  399
427H      KGAKIIIPAAAPSTPTNSTKNEDSAPTEHVDRGIATNETQVEVGIDADFDGLLEATEAAEVKSRHQRRAMIILAVLVPAI  397
1125C     KGAKIIIPAAAPSTPTNSTKNEDSAPTEHVDRGIATNETQVEVGIDADFDGLLEATEAAEVKSRHQRRAMIILAVLVPAI  397
427I      KGAKIIIPAAAPSTPTNSTKNEDSAPTEHVDRGIATNETQVEVGIDADFDGLLEATEAAEVKSRHQRRAMIILAVLVPAI  397
1125A     KGAKIIIPAAAPSTPTNSTKNEDSAPTEHVDRGIATNETQVEVGIDADFDGLLEATEAAEVKSRHQRRAMIILAVLVPAI  397
427J      KGAKIIIPAAAPSTPTNSTKNEDSAPTEHVDRGIATNETQVEVGIDADFDGLLEATEAAEVKSRHQRTAIIILAVLVPAI  397
927E      KGAKIIIPAAAPATPTNSTKNEDSASTEHVDRGIATNETQVEVGIDADFDSLLDATEAAEVTRRHQRTAMIILAVLVPAI  397
927B      KGAKIIIPAAAPATPTNSTKNEDSASTEHVDRGIATNETQVEVGIDADFDSLLDATEAAEVTRRHQRTAMIILAVLVPAI  397
927C      KGAKIIIPAAAPATPTNSTKNEDSASTEHVDRGIATNETQVEVGIDADFDSLLDATEAAEVTRRHQRTAMIILAVLVPAI  397
927D      KGAKIIIPAAAPATPTNSTKNEDSASTEHVDRGIATNETQVEVGIDADFDSLLDATEAAEVTRRHQRTAMIILAVLVPAI  397
927A      KGAKIIIPAAAPATPTNSTKNEDSASTEHVDRGIATNETQVEVGIDADFDSLLDATEAAEVTRRHQRTAMIILAVLVPAI  397
427C      KGAKIIIPAAAPVTPTNSTKNEDSAPTEHVDRGIATNETQVEVGIDADFDGLLEAAEAAEVTRRHQKTAMIILAVLVPAI  397
1125D     KGAKIIIPAAAPVTPTNSTKNEDSAPTEHVDRGIATNETQVEVGIDADFDSLLDATEAAEVTRRHQRTAMIILAVLVPAI  397
427G      KGAKIIIPAAAPVTPTNSTKNEDSAPTEHVDRGIATNETQVEVGIDADFDSLLDATEAAEVTRRHQRTAMIILAVLVPAI  397
427B      KGAKIIIPAAAPVTPTNSTKNEDSAPTEHVDRGIATNETQVEVGIDADFDSLLDATEAAEVTRRHQRTAMIILAVLVPAI  397
427D      KGAKIIIPAAAPVTPTNSTKNEDSAPTEHVDRGIATNETQVEVGIDADFDGLLEAAEAAEVTRGHQKTAMIILAVLVPAI  397
427E      KGAKIIIPAAAPVTPTNSTKNEDSAPTEHVDRGIATNETQVEVGIDADFDGLLEAAEAAEVTRGHQKTAMIILAVLVPAI  397
427A      KGAKIIIPAAAPVTPTNSTKNEDSAPTEHVDRGIATNETQVEVGIDADFDGLLEAAEAAEVTRRHQRTAMIILAVLVPAI  397
1125F     KGAKIIIPAAAPVTPTNSTKNEDSAPTEHVDRGIATNETQVEVGIDADFDGLLEAAEAAEVTRRHQKTAMIILAVLVPAI  397
1125B     KGAKIIIPAAAPVTPTNSTKNEDSAPTEHVDRGIATNETQVEVGIDADFDGLLEAAEAAEVTRGHQKTAMIILAVLVPAI  397
1125G     KGAKIIIPAAAPVTPTNSTKNEDSAPTEHVDRGIATNETQVEVGIDADFDGLLEAAEAAEVTRRHQKTAMIILAVLVPAI  397
427F      KGAKIIIPAAAPVTPTNSTKNEDSAPTEHVDRGIATNETQVEVGIDADFDGLLEAAEAAEVTRRHQKTAMIILAVLVPAI  397

                      410         420       430
1125E     ILVVTAVAFFIMVKRRRNNSQDVDTGKAEGGVSSGKVVM                                           438
427K      ILVVTAVAFFIMVKRRRNNSQDVDTGKAEGGVSSGKVVM                                           438
927F      ILA-AAVAFFIMVKRRRNSSQDVDTGKAEGGVSSVKVVM                                           437
427H      ILVVTAVAFFIMVKRRRNNSQDVDTGKAEGGVSSGKAVM                                           436
1125C     ILVVTAVAFFIMVKRRRNNSQDVDTGKAEGGVSSGKAVM                                           436
427I      ILVVTAVAFFIMVKRRRNNSQDVDTGKAEGGVSSGKAVM                                           436
1125A     ILVVTAVAFFIMVKRRRNNSQDVDTGKAEGGVSSGKAVM                                           436
427J      ILVVTAVAFFIMVKRRRNNSQDVDTGKAEGGVSSGKAVM                                           436
927E      ILVVTAVAFFIMVKRRRNSSQDVDTGKAEGGVSSVKVVM                                           436
927B      ILVVTAFAFFIMVKRRRNSSKDVDTGKAEGGVSSVKVVM                                           436
927C      ILVVTAVAFFIMVKRRRNSSKDVDTGKAEGGVSSVKVVM                                           436
927D      ILVVTAVAFFIMVKRRRNSSKDVDTGKAEGGVSSVKVVM                                           436
927A      ILVVTAVAFFIMVKRRRNSSKDVDTGKAEGGVSSVKVVM                                           436
427C      ILVVTAVAFFIMVKRRRNNSRDVDTGKAEGGVSSVKVVM                                           436
1125D     ILVVTAVAFFIMVKRRRNSSKDVDTGKAEGGVSSVKVVM                                           436
427G      ILVVTAVAFFIMVKRRRNSSKDVDTGKAEGGVSSVKVVM                                           436
427B      ILVVTAVAFFIMVKRRRNSSKDVDTGKAEGGVSSVKVVM                                           436
427D      ILVVTAVAFFIMVKRRRNNSRDVDTGKAEGGVSSVKVVM                                           436
427E      ILVVTAVAFFIMVKRRRNNSRDVDTGKAEGGVSSVKVVM                                           436
427A      ILVVTAVAFFIMVKRRRNNSRDVDTGKAEGGVSSVKVVM                                           436
1125F     ILVVTAVAFFIMVKRRRNNSQDVDTGKAEGGVSSVKVVM                                           436
1125B     ILVVTAVAFFIMVKRRRNNSQDVDTGKAEGGVSSVKVVM                                           436
1125G     ILVVTAVAFFIMVKRRRNNSQDVDTGKAEGGVSSVKVVM                                           436
427F      ILVVTAVAFFIMVKRRRNNSQDVDTGKAEGGVSSVKVVM                                           436
```

Supplementary Table 1: Mass spectrometry analysis identifies complement C3 as the ligand pulled down by ISG65

Pulldown samples were analysed by SDS-PAGE and a region of the gel containing two proteins unique to ISG65 were excised, as well as the corresponding region from a FHR pulldown from bovine serum as a control (Fig. 1c). Samples were trypsin-digested and analyzed by ESI-TRAP. All top hits are listed, except keratin which was removed from the list as a contaminant. This data is representative of two independent pulldowns and mass spectrometry analyses. Enrichment of proteins in ISG65 compared to FHR was performed.

**ISG65 enriched or unique hits**									
**Protein**		**MW**	**Peptides[2]**	**Coverage (%)[3]**	**Spectral counts[4]**		**Spectral index[5]**		**Ratio[6]**
**Accession #[1]**	**Description**	**(kDa)**			**ISG65**	**FHR**	**ISG65**	**FHR**	**ISG65/FHR**
Q2UVX4	Complement C3	187.62	92	50.1	91.97	12.00	5.01^{-4}	4.78^{-5}	10.48
A6QPP2	SERPIND1 protein	55.32	6	12.9	1.00	n/a	3.38^{-7}	n/a	ISG65 only
G3N0V0	Uncharacterized protein	36.02	5	16.3	1.00	n/a	1.86^{-6}	n/a	ISG65 only
P06868	Plasminogen	91.42	4	4.2	2.97	n/a	2.78^{-6}	n/a	ISG65 only
F1MCF8	Uncharacterized protein	24.44	2	9.8	1.00	n/a	9.14^{-7}	n/a	ISG65 only
G5E5T5	Uncharacterized protein	42.55	3	8.2	1.00	n/a	1.26^{-6}	n/a	ISG65 only

[1]Accession number on UnitProt.
[2]Number of peptide sequences.
[3]Percent coverage of the protein with the found peptides.
[4]Spectral counts have been improved on the CPFP server by including fragment ion intensities in their caluation of [5]their spectral index.
[6]The spectral index is used to calculate the enrichment ratio. Complement C3 is enriched 10.5-fold in the ISG65 sample compared to the FHR sample.

Supplementary Table 2: ISG65 pulls down complement C3 from a broad spectrum of mammalian species

Pull-down samples the sera of various mammals were analysed by SDS-PAGE, and the most intense band excised for trypsin-digest mass spectrometry analysis (Figure 1d). The top hit from the corresponding species is listed, except keratin which was removed from the list as a contaminant.

**Species**	**Accession #[1]**	**Description**	**Score**	**Peptides[2]**	**Coverage (%)[3]**	**PSM[4]**
Human	P01024	Complement C3	1739	64	100	1015
Cow	Q2UVX4	Complement C3	1002	49	85	449
Horse	A0A3Q2HWQ6	Complement C3	158	11	17	102
Goat	A0A452DXE2	Complement C3	353	17	26	219
Dog	F1MCF8	Complement C3	845	46	87	715
Rabbit	P12247	Complement C3 alpha chain	105	21	17	96

[1]Accession number on UnitProt.
[2]Number of peptide sequences.
[3]Percent coverage of the protein with the found peptides
[4]Peptide spectrum matches

Supplementary Table 3: Kinetic parameters measured by surface plasmon resonance

Average values over three experimental repeats with corresponding standard deviation are displayed. For C3b and C3c, replicates were performed on three different flow paths with different immobilisation levels of ISG65. For C3d, replicates were performed on the same flow path.

	k_{a1} ($M^{-1}s^{-1}$)	k_{d1} (s^{-1})	k_{a2} (s^{-1})	k_{d2} (s^{-1})	K_D (nM)	Chi^2 (RU^2)
C3b	$9 \times 10^4 \pm 2 \times 10^4$	0.06 ± 0.009	0.01 ± 0.0004	0.001 ± 0.0005	67 ± 21	25 ± 25
C3c	$2 \times 10^4 \pm 1 \times 10^4$	0.008 ± 0.004	-	-	391 ±54	3.5 ± 3.9
C3d	$3 \times 10^5 \pm 7 \times 10^5$	0.1 ± 0.03	-	-	514 ± 90	5.2 ± 2.5

Supplementary Table 4: crystallographic statistics

**Data collection**	
Space group	$P12_11$
Cell dimensions:	
a, b, c (Å)	51.95, 189.58, 73.88
α, β, γ (°)	90.00, 90.11, 90.00
Resolution (Å)	94.79 – 2.6 (2.70-2.60)
Total observations	125,121 (13,131)
Total unique	43,218 (4392)
R_{pim}	0.165 (0.954)
$CC_{1/2}$	0.875 (0.31)
I/σ(I)	3.6 (0.7)
Completeness (%)	98.7 (96.4)
Multiplicity	2.9 (3.0)
Wilson B factor ($Å^2$)	41.0
**Refinement**	
Number of reflections	43141
R_{work} / R_{free}	0.2698 / 0.3100
Average B factor ($Å^2$)	51.0
Number of residues:	
Amino acid residues	996
Ligand molecules (GOL / PG0)	6
Waters	60
RMSZ deviations	
Bond lengths	0.007
Bond angles	0.900
Ramachandran plot	
Favoured (%)	96.4
Allowed (%)	3.5
Outliers (%)	0.1

Supplementary Table 5: Interactions between ISG65 and C3d

**ISG65 residue**	**Atom**	**C3d residue**	**Atom**
Hydrogen bonds			
D1174	OD2	Y70	OH
E1110	OE2	K84	NZ
E1110	OE2	N296	ND2
E1110	OE2	W81	NE1
E1110	OE2	Y80	OH
I1108	O	R77	NH2
K1111	O	Y293	OH
K1171	NZ	E74	OE2
L1109	O	R77	NH2
Q1112	O	R77	NH2
Q1112	O	R77	NH1
Q1119	OE1	Y293	OH
T1170	OG1	Y70	OH
Salt bridges			
K1171	NZ	E74	OE2
K1203	NZ	E59	OE1
E1110	OE2	K84	NZ
Residue-residue			
D1121		Q301	
D1121		T297	
E1110		N296	
E1110		Y293	
G1204		Y209	
I1108		F73	
K1111		Y293	
K1113		Y293	
K1171		F73	
K1171		Y70	
L1109		F73	
L1109		R77	
L1109		W81	
L1109		Y76	
L1109		Y80	
P1114		A289	
P1114		L286	
P1114		N290	
P1114		N290	
P1114		Y293	
P1205		Y209	
Q1119		Y293	
R1201		R63	
S1164		F73	

Uncropped blots for supplementary figures

Uncropped blot of RNAi knockdown of ISG65 in supplementary figure 1b.

A northern blot probed for ISG65. For more information, see Supplementary Figure 1b

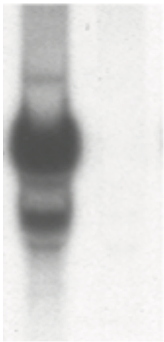

A northern blot probed for rRNA. For more information, see Supplementary Figure 1b

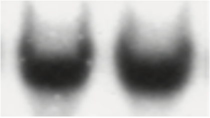

Uncropped blot of RNAi knockdown of ISG65 in supplementary figure 2c.

A Western blot using a polyclonal sera targeting ISG65 for detection. For more information, see Supplementary Figure 2c

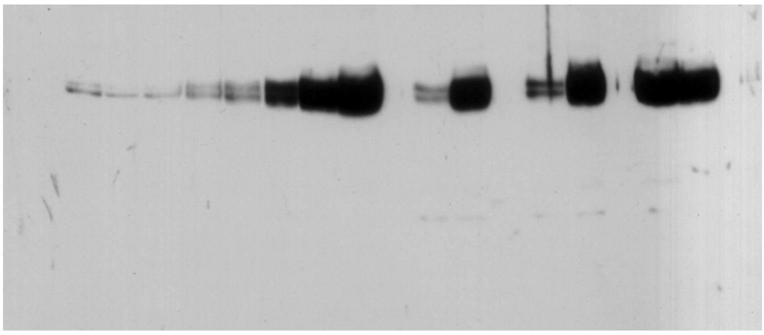

Supplement: Supplementary file 1 — Supplementary information [file 41467_2022_32728_MOESM1_ESM.pdf]
