# Supplementary material for: Invariant surface glycoprotein 65 of Trypanosoma brucei is a complement C3 receptor
Source: Nat Commun. 2022 Aug 29;13:5085. doi: 10.1038/s41467-022-32728-9 (PMC9424271; doi:10.1038/s41467-022-32728-9)
Supplement: Supplementary file 2 — Description of Additional Supplementary Information [file 41467_2022_32728_MOESM2_ESM.pdf]

Description of additional supplementary files

Supplementary data 1: ISG65 sequences

Sequences for each of the ISG65 genes in the EATRO1125, TREU927 and Lister 427 strains from *T. b. brucei*.

Supplementary data 2: SAXS data

Raw scattering data for small angle x-ray scattering analysis of the ISG65-C3d complex.
