# Supplementary material for: Invariant surface glycoprotein 65 of Trypanosoma brucei is a complement C3 receptor
Source: Nat Commun. 2022 Aug 29;13:5085. doi: 10.1038/s41467-022-32728-9 (PMC9424271; doi:10.1038/s41467-022-32728-9)
Supplement: Supplementary file 5 — Reporting Summary [file 41467_2022_32728_MOESM5_ESM.pdf]

nature portfolio

Corresponding author(s): Mark Carrington; Matthew K. Higgins

Last updated by author(s): Jul 21, 2022

Reporting Summary

Nature Portfolio wishes to improve the reproducibility of the work that we publish. This form provides structure for consistency and transparency in reporting. For further information on Nature Portfolio policies, see our Editorial Policies and the Editorial Policy Checklist.

Statistics

For all statistical analyses, confirm that the following items are present in the figure legend, table legend, main text, or Methods section.

n/a	Confirmed	
☐	☒	The exact sample size (*n*) for each experimental group/condition, given as a discrete number and unit of measurement
☒	☐	A statement on whether measurements were taken from distinct samples or whether the same sample was measured repeatedly
☒	☐	The statistical test(s) used AND whether they are one- or two-sided<br>*Only common tests should be described solely by name; describe more complex techniques in the Methods section.*
☒	☐	A description of all covariates tested
☒	☐	A description of any assumptions or corrections, such as tests of normality and adjustment for multiple comparisons
☐	☒	A full description of the statistical parameters including central tendency (e.g. means) or other basic estimates (e.g. regression coefficient) AND variation (e.g. standard deviation) or associated estimates of uncertainty (e.g. confidence intervals)
☒	☐	For null hypothesis testing, the test statistic (e.g. *F*, *t*, *r*) with confidence intervals, effect sizes, degrees of freedom and *P* value noted<br>*Give P values as exact values whenever suitable.*
☒	☐	For Bayesian analysis, information on the choice of priors and Markov chain Monte Carlo settings
☒	☐	For hierarchical and complex designs, identification of the appropriate level for tests and full reporting of outcomes
☒	☐	Estimates of effect sizes (e.g. Cohen's *d*, Pearson's *r*), indicating how they were calculated

Our web collection on statistics for biologists contains articles on many of the points above.

Software and code

Policy information about availability of computer code

Data collection	Data collection and processing software used are described in the methods section and are commercially available or openly accessible.
Data analysis	Data analysis was performed as described in the methods section using commercially available or openly accessible software. Axiovision (Zeiss) was used to analyse immunofluorescence images. The Mascot search engine and SEQUEST HT within Proteome discoverer PD1.4 (Thermo Fischer Scientific, version 1.4.0.288) were used for mass spectrometry searches. BIAevaluate v1.0 was used for SPR analysis. AIMLESS v0.7.7, CCP4i2 v1.0.2, PHASER v2.8.3, BUSTER v2.1, Coot v0.9.4, ChimeraX v1.1 were used for crystallography analysis. ATSAS v3.0.3 and DENSS v1.6 were used for SAXS analysis. Software used for crystallographic data processing and structure determination and SAXS analysis is the standard in the field and is all freely available to academic users. Trimmomatic, Bowtie2, and Artemis were used for genome analysis. GraphPad Prism version 7 was used to generate graphs, PyMOL for structure visualisation, and Affinity Photo and Designer and Adobe Photoshop and Illustrator to generate figures.

For manuscripts utilizing custom algorithms or software that are central to the research but not yet described in published literature, software must be made available to editors and reviewers. We strongly encourage code deposition in a community repository (e.g. GitHub). See the Nature Portfolio guidelines for submitting code & software for further information.

Data

Policy information about availability of data

All manuscripts must include a data availability statement. This statement should provide the following information, where applicable:

- Accession codes, unique identifiers, or web links for publicly available datasets
- A description of any restrictions on data availability
- For clinical datasets or third party data, please ensure that the statement adheres to our policy

The T. brucei EATRO1125 ISG65 A to G gene sequences used in this study have been deposited in the European Nucleotide Archive under accession codes OU529038-OU529044. The raw reads from the genome sequencing used in this study have been deposited in the ArrayExpress database under accession code E-MTAB-10878. Coordinates and structure factors used in this study have been deposited in the Protein Data Bank under accession code 7PI6. Un-cropped gels generated in this study are provided as source datafiles 1-3. Source data for all graphs generated in this study are provided as source datafile 4.xlsx. Raw SAXS data generated in this study is provided in source data file 5.zip.

Field-specific reporting

Please select the one below that is the best fit for your research. If you are not sure, read the appropriate sections before making your selection.

- [x] Life sciences
- [ ] Behavioural & social sciences
- [ ] Ecological, evolutionary & environmental sciences

For a reference copy of the document with all sections, see nature.com/documents/nr-reporting-summary-flat.pdf

Life sciences study design

All studies must disclose on these points even when the disclosure is negative.

Sample size	The sample sizes for mouse infection experiments are the standard in the field (most reports use n=3-6 per group for infection dynamics) and were determined based on the sample sizes required to see differences in infection dynamics in previous experiments. Bioluminescence provides a more statistically robust measure of parasite burden than other methods such as PCR or counting trypomastigotes in a hemocytometer as it provides a measure of the burden from all tissues, not just the bloodstream, making these sample sizes apporpriate.
Data exclusions	No data were excluded from analysis.
Replication	All SPR experiments were repeated three times. Assessments of trypanosome infection were performed in five mice for each variant of trypanosomes and mice. All attempts at replication were successful.
Randomization	No randomisation was conducted as no decisions about inclusion or exclusion of data were taken. All data was included in analysis.
Blinding	No blinding was conducted. This was not required as each experiment was designed to give an unambiguous outcome which did not depend on the judgement of the researcher or the selection of which images or cells to include in analysis.

Reporting for specific materials, systems and methods

We require information from authors about some types of materials, experimental systems and methods used in many studies. Here, indicate whether each material, system or method listed is relevant to your study. If you are not sure if a list item applies to your research, read the appropriate section before selecting a response.

Materials & experimental systems

n/a	Involved in the study
[ ]	[x] Antibodies
[ ]	[x] Eukaryotic cell lines
[x]	[ ] Palaeontology and archaeology
[ ]	[x] Animals and other organisms
[x]	[ ] Human research participants
[x]	[ ] Clinical data
[x]	[ ] Dual use research of concern

Methods

n/a	Involved in the study
[x]	[ ] ChIP-seq
[x]	[ ] Flow cytometry
[x]	[ ] MRI-based neuroimaging

Antibodies

Antibodies used	The ISG65 antiserum was produced by Covalab, as described in this study and its production and validation are described in the methods section. It was used at 1 in 8,000 dilution.<br>Anti-eIF4A was generated as described in Dhalia R et al. 'The two eIF4A helicases in Trypanosoma brucei are functionally distinct. Nucleic Acids Res. 2006 May 10;34(9):2495-507 (doi: 10.1093/nar/gkl290)' and was used at 1 in 6,000 dilution.

	Mouse monoclonal anti-FAZ loading control was a kind gift of Keith Gull, University of Oxford (Assembly of the paraflagellar rod and the flagellum attachment zone complex during the Trypanosoma brucei cell cycle L Kohl 1, T Sherwin, K Gull PMID: 10361731 DOI: 10.1111/j.1550-7408.1999.tb04592.x) and was used at 1 in 200 dilution. The remaining secondary antibodies are commerically available: donkey and goat anti-rabbit horseradish peroxidase conjugates (Jackson labs 711-035-152 and 111-035-003 respectively) were used at 1 in 10,000 dilution; donkey anti-rabbit IgG AlexaFluor 488 (Thermofisher A-32790) was used at dilution 1 in 1,000, and goat anti-mouse IgG AlexaFluor 568 (Thermofisher A-11004) was used at dilution 1 in 1,000.
Validation	All antibodies were validated within this work as described in the methods and shown in Figures 1a and 1b.

Eukaryotic cell lines

Policy information about cell lines

Cell line source(s)	Trypanosoma brucei brucei EATRO1125 was provided by Etienne Pays as a procyclic cell line (ProG) and was differentiated to bloodstream forms by passage through tsetse flies (The GPI-phospholipase C of Trypanosoma brucei is nonessential but influences parasitemia in mice. (Webb H, Carnall N, Vanhamme L, Rolin S, Van Den Abbeele J, Welburn S, Pays E, Carrington M. J Cell Biol. 1997 Oct 6;139(1):103-14. doi: 10.1083/jcb.139.1.103). HEK293 cells and CHO cells (Thermofisher) were commercially available and were only used as expression systems for protein production and not studied as cellular systems.
Authentication	Cell lines (HEK293 and CHO) used for protein expression were not authenticated. Trypanosome cell lines were authenticated by genome sequencing.
Mycoplasma contamination	Mycoplasma contamination not applicable and mycoplasma has not been seen to infect trypanosome cultures
Commonly misidentified lines (See ICLAC register)	No commonly misidentified cell lines were used in this work.

Animals and other organisms

Policy information about studies involving animals; ARRIVE guidelines recommended for reporting animal research

Laboratory animals	All experiments were performed using female BALB/c 6-8 weeks of age, female C57BL/6J 8-9 weeks of age, both purchased from Charles River (UK), and female B6;129S4-C3tm1Crr/J mice 8-9 weeks of age purchased from the Jackson Laboratory (USA) via Charles River (UK). Additional information is provided in the methods.
Wild animals	The study did not involve wild animals.
Field-collected samples	The study did not involve samples collected from the field.
Ethics oversight	Research was carried out under UK Home Office project licenses PPL 70/8207 and P9AEE04E4, with approval of the LSHTM Animal Welfare and Ethical Review Board, and in accordance with the UK Animals (Scientific Procedures) Act 1986 (ASPA).

Note that full information on the approval of the study protocol must also be provided in the manuscript.
